# Combined Approach for Government E-Tendering Using GA and TOPSIS with Intuitionistic Fuzzy Information

**DOI:** 10.1371/journal.pone.0130767

**Published:** 2015-07-06

**Authors:** Yan Wang, Chengyu Xi, Shuai Zhang, Wenyu Zhang, Dejian Yu

**Affiliations:** School of Information, Zhejiang University of Finance and Economics, Hangzhou, China; Nankai University, CHINA

## Abstract

As E-government continues to develop with ever-increasing speed, the requirement to enhance traditional government systems and affairs with electronic methods that are more effective and efficient is becoming critical. As a new product of information technology, E-tendering is becoming an inevitable reality owing to its efficiency, fairness, transparency, and accountability. Thus, developing and promoting government E-tendering (GeT) is imperative. This paper presents a hybrid approach combining genetic algorithm (GA) and Technique for Order Preference by Similarity to an Ideal Solution (TOPSIS) to enable GeT to search for the optimal tenderer efficiently and fairly under circumstances where the attributes of the tenderers are expressed as fuzzy number intuitionistic fuzzy sets (FNIFSs). GA is applied to obtain the optimal weights of evaluation criteria of tenderers automatically. TOPSIS is employed to search for the optimal tenderer. A prototype system is built and validated with an illustrative example from GeT to verify the feasibility and availability of the proposed approach.

## Introduction

Tendering, since its introduction, has been considered to be one of the most impartial means of awarding government contracts and the method with the highest expectation of securing a favorable outcome for a government spending public funds [1–2]. Compared with traditional tendering, E-tendering combines conventional tendering with Internet, computer, and certification technologies to realize the electronization and informatization of tendering process. Nowadays, because of the development of Internet, information, and communication technologies, E-tendering is gaining popularity in enterprises owing to its efficiency, fairness, transparency, and accountability.

Numerous governments are establishing E-tendering systems for procuring building services and goods [3]. Compared with conventional government tendering, government E-tendering (GeT) (1) promotes the informatization of enterprises and accelerates the integration of enterprises into the international supply chain; (2) It can reduce the costs of both tenderees and tenderers, save social resources, and improve the efficiency of tendering procedures; (3) Moreover, it can standardize market order, alleviate corruption, and establish an impartial and transparent tendering environment. Therefore, developing and promoting an efficient and effective GeT system is a critical and timely task.

Developing an efficient and effective GeT system to exploit the advantages of GeT to the utmost is an intricate project requiring significant functionality including web-based bid evaluation, which is an essential requirement of the E-tendering procedure. However, in current literature, there still has minimal attention been addressed to web-based bid evaluation. In this paper, we propose a novel and hybrid approach for web-based bid evaluation of GeT searching for the optimal tenderer efficiently and fairly under circumstance where the attributes of the tenderers are expressed as fuzzy number intuitionistic fuzzy sets (FNIFSs). Owing to the refinement and objectivity of FNIFS in describing objective items, we employ it to describe the tenderers' attributes so as to ensure the accuracy of the search results. Genetic algorithm (GA) and Technique for Order Preference by Similarity to an Ideal Solution (TOPSIS) are applied to obtain the optimal weights of the evaluation criteria of the tenderers and identify the ideal tenderer from a finite set of qualified candidate tenderers, respectively.

The remainder of this paper is organized as follows: Section 2 presents related works. An overview of the proposed approach is summarized in Section 3. This contains two parts: weight optimization and optimal tenderer identification. Sections 4 and 5 elaborate these two functions, respectively. An illustrative example with a prototype system is built and validated in Section 6. Section 7 concludes this paper.

## Related Works

### A. Government E-tendering

The past decade has seen the rapid development of E-government that is strongly epitomized by a general agreement among E-government scholars that governments are ready to enter a transactions-based phase of E-government development [4–6]. Conventional government tendering, as an essential government procurement method, has always been regarded as the most impartial and equitable procurement method for transacting with vendors and enterprises. However, traditional tendering process is paper-based and involves significant manual effort that can create difficulties [7]. Furthermore, preparing tendering documentation and conducting the tendering process requires intensive labor [8]. Thus, the evolution of government tendering to electronization is inevitable.

As E-tendering begins to gain acceptance from governments because of its obvious and abundant benefits, numerous GeT systems are beginning to appear. Unfortunately, problems have arisen along with these GeT systems: the lack of corresponding laws and regulations, the lack of interoperation among functions and information of GeT systems, and unsuitable administrative supervision. Although there has been minimal research on GeT, in recent years, numerous studies addressing E-tendering have been undertaken with incremental interest in E-tendering by scholars focusing on methods to assess the performance of E-tendering, the critical factors to be considered in implementing a successful E-tendering system, and procedures to address the problems identified above. These studies provide constructive proposals for the implementation and promotion of GeT. Chu *et al*. [9] explored the critical success factors of Taiwan's GeT system through the behavioral perspectives of the end users. Lou and Alshawi [10] and Masher *et al*. [11] presented available solutions aimed at resolving several problems of the implementation of E-tendering in the construction industry. Mondorf and Wimmer [12] introduced findings from existing studies for designing a Virtual Company Dossier, a key building block of E-tendering systems. Du [13] proposed an automatic E-tendering system that implements an automatic negotiation process over the Semantic Web.

However, there has been no study proposing an approach to evaluate tenderers' personal attributes automatically and efficiently. This paper presents a novel and hybrid approach for GeT using both GA and TOPSIS. GA can be easily interfaced to existing models and simulations [14]. TOPSIS is an excellent multiple criteria method to identify solutions from a finite set [15] where the tenderers' attributes are expressed as FNIFSs. FNIFSs are employed because of their refinement and objectivity in describing items and strength of expressing tenderers' attributes as precise values.

### B. Fuzzy number intuitionistic fuzzy set

The key information required in a multi-attribute decision-making (MADM) model includes attribute values, attribute weights, and a mechanism to synthesize this information into an aggregated value or assessment for each alternative [16]. However, in the GeT process, the tenderers' individual attributes are always uncertain, imprecise, and vague by nature. Thus, it is difficult for experts to provide their evaluations on tenderers' attributes in precise values. Therefore, we use FNIFSs to express tenderers' attributes accurately and objectively.

Fuzzy set theory was proposed by Zadeh [17] in 1965 and has been implemented in successful applications in numerous fields in the past decades. Atanassov [18] extended fuzzy set theory and introduced the concept of intuitionistic fuzzy set (IFS), defined in the following.

**Definition 2.1** An IFS *A* in *X* is given by:

$$A=\left\{\langle x,\mu_{A}\left(x\right),\nu_{A}\left(x\right)\rangle |x\in X\right\},$$ (1)

where *μ*_*A*_ *(x)*: *X →*[0,1] and *ν*_*A*_ *(x)*: *X →*[0,1], with the condition:

$$0\leq \mu_{A}\left(x\right)+\nu_{A}\left(x\right)\leq 1,\forall x\in X,$$ (2)

The numbers *μ*_*A*_ *(x)* and *ν*_*A*_ *(x)* represent the membership and non-membership degree of the element *x* to the set *A*, respectively. For each IFS *A* in *X*, let *π*_*A*_ *(x) = 1 - μ*_*A*_ *(x)—ν*_*A*_ *(x)*, then *π*_*A*_ *(x)* is called the indeterminacy degree of element *x* to set *A*.

FNIFS is a generalization of IFS that extends the IFS theory with fuzzy number theory. FNIFS has the same form as IFS, while further fuzzifies IFS. Liu and Yuan [19] introduced the concept of FNIFS, described as follows.

**Definition 2.2** Let *X* be a universe of discourse, an FNIFS *A* ^*^ over *X* is an object having the form:

$$A^{*}=\left\{\langle x,\mu_{A}^{*}\left(x\right),\nu_{A}^{*}\left(x\right)\rangle |x\in X\right\},$$ (3)

where *μ* ^*^_A_ *(x) = (μ* ^*L^_*A*_ *(x)*, *μ* ^*M^_*A*_ *(x)*, *μ* ^*H^_*A*_ *(x)) (0<μ* ^*L^_*A*_ *(x)<μ* ^*M^_*A*_ *(x)<μ* ^*H^_*A*_ *(x)<1)* and *ν* ^*^_A_ *(x) = (ν* ^*L^_*A*_ *(x)*, *ν* ^*M^_*A*_ *(x)*, *ν* ^*H^_*A*_ *(x)) (0<ν* ^*L^_*A*_ *(x)<ν* ^*M^_*A*_ *(x)<ν* ^*H^_*A*_ *(x)<1)* are two triangular fuzzy numbers in the interval [0,1], with the condition:

$$\mu_{A}^{*H}\left(x\right)+\nu_{A}^{*H}\left(x\right)\leq 1,\forall x\in X,$$ (4)

**Definition 2.3** Let *α*_*1*_ *= ‹(a*_*1*_,*b*_*1*_,*c*_*1*_ *)*,*(l*_*1*_,*m*_*1*_,*p*_*1*_ *)›* and *α*_*2*_ *= ‹(a*_*2*_,*b*_*2*_,*c*_*2*_ *)*,*(l*_*2*_,*m*_*2*_,*p*_*2*_ *)›* be two FNIFSs, then:

$$\alpha_{1}+\alpha_{2}=\langle \left(a_{1}+a_{2}-a_{1}a_{2},b_{1}+b_{2}-b_{1}b_{2},c_{1}+c_{2}-c_{1}c_{2}),(l_{1}l_{2},m_{1}m_{2},p_{1}p_{2}\right)\rangle ;$$

$$\alpha_{1}\times \alpha_{2}=\langle \left(a_{1}a_{2},b_{1}b_{2},c_{1}c_{2}),(l_{1}+l_{2}-l_{1}l_{2},m_{1}+m_{2}-m_{1}m_{2},p_{1}+p_{2}-p_{1}p_{2}\right)\rangle ;$$

$$\lambda \alpha_{1}=\langle \left(1-{\left(1-a_{1}\right)}^{\lambda },1-{\left(1-b_{1}\right)}^{\lambda },1-{\left(1-c_{1}\right)}^{\lambda }),(l_{1}{}^{\lambda },m_{1}{}^{\lambda },p_{1}{}^{\lambda }\right)\rangle ;$$

$$\alpha_{1}{}^{\lambda }=\langle \left(a_{1}{}^{\lambda },b_{1}{}^{\lambda },c_{1}{}^{\lambda }),(1-{\left(1-l_{1}\right)}^{\lambda },1-{\left(1-m_{1}\right)}^{\lambda },1-{\left(1-p_{1}\right)}^{\lambda }\right)\rangle ,\lambda \geq 0,$$

Chen and Tan [20] proposed the IFS score function that allows the membership and non-membership degree of each alternative to be expressed as vague values. Then, Hong and Choi [21] introduced the IFS accuracy function, because, in some cases, the score function cannot provide adequate information for the alternatives.

**Definition 2.4** Let *α = ‹μ*_*α*_,*ν*_*α*_ *›* be an IFS, the score function of *α* can be represented as:

$$S(\alpha )=\mu_{\alpha }-\nu_{\alpha },\;S(\alpha )\in \left[-1,1\right],$$ (5)

and the accuracy function of *α* can be represented as:

$$H(\alpha )=\mu_{\alpha }+\nu_{\alpha },\;H(\alpha )\in \left[0,1\right],$$ (6)

Subsequently, based on the score and accuracy functions, Xu and Yager [22] proposed the following comparison rules for two IFSs.

**Definition 2.5** Let *α*_*1*_, *α*_*2*_ be two IFSs, *S(α*_*1*_ *)* and *S(α*_*2*_ *)* be the score function of *α*_*1*_ and *α*_*2*_, respectively, and *H(α*_*1*_ *)* and *H(α*_*2*_ *)* be the accuracy function of *α*_*1*_ and *α*_*2*_, respectively. Then, if *S(α*_*1*_ *)<S(α*_*2*_ *)*, *α*_*1*_ *<α*_*2*_; If *S(α*_*1*_ *) = S(α*_*2*_ *)*, then (1) if *H(α*_*1*_ *)<H(α*_*2*_ *)*, *α*_*1*_ *<α*_*2*_; (2) If *H(α*_*1*_ *) = H(α*_*2*_ *)*, *α*_*1 =*_ *α*_*2*_.

Then, Wang [23] proposed the concept of the score and accuracy functions of a FNIFS.

**Definition 2.6** Let *α = ‹(a*,*b*,*c*_*)*_,*(l*,*m*,*p*_*)*_ *›* be a FNIFS, the score function of *α* can be represented as:

$$S^{*}(\alpha )=\frac{a+2b+c}{4}-\frac{l+2m+p}{4},S^{*}(\alpha )\in \left[-1,1\right],$$ (7)

and the accuracy function of *α* can be represented as:

$$H^{*}(\alpha )=\frac{a+2b+c}{4}+\frac{l+2m+p}{4},H^{*}(\alpha )\in \left[0,1\right],$$ (8)

The comparison rules for two FNIFSs are the same as those for two IFSs.

The past decades have seen an exponential growth of research on IFSs, whereas research on FNIFS remains rare. To begin, this paper investigates the potential of employing FNIFS theory for the optimal selection of candidate tenderers in the GeT process. FNIFS theory is combined with GA and TOPSIS. The FNIFS-based GA is used to generate the weight information of evaluation criteria. The FNIFS-based TOPSIS is applied to identify the optimal tenderer from all candidate tenderers.

### C. Genetic algorithm

GA was proposed by Holland [24] in the 1970's and contributes to numerous scientific and engineering applications. It is a computation module that imitates the biological evolution process of natural selection and the genetic mechanism of Darwin's biological theory of evolution. It employs a probability-based optimization method and can automatically adjust the search direction without a pre-established rule. These advantageous properties enable GA to exhibit a superior ability for global optimization and to be widely applied to multiple fields [25–30] including combinatorial optimization, machine learning, signal processing, adaptive control, and artificial life.

In this paper, we receive a set of the optimal weights of the evaluation criteria of tenderers automatically, i.e., the system allocates one weight to each evaluation criterion of the tenderers and these weights reflect precisely the degree of importance of each of the evaluation criteria. However, standard GA has limitations as a problem becomes overly complicated. Thus, we have made appropriate modifications to the chromosomes, operators, and implementation. These include a new fitting function that is suitable for addressing the intuitionistic fuzzy information, the real-value chromosome representation scheme, the selection operator that can randomly generate the initial population, the uniform crossover operator, and a new three-time mutation operator.

### D. TOPSIS

TOPSIS was proposed by Hwang and Yoon [31] in 1981 and has been widely studied and developed in numerous fields in recent years [32–38]. This process sequences alternatives according to the order of closeness degree between target and ideal alternatives. It optimizes the original data and eliminates the influence generated by diverse metrics. This allows TOPSIS to comprehensively reflect and evaluate the overall situation.

In this paper, we adopt TOPSIS as an effective and efficient approach to identify the optimal tenderer from all candidate tenderers for its authenticity, understandability, and reliability; it has no particular requirement for sample data. Furthermore, because of the special demands of the scenarios in this paper, we propose a modified FNIFS-based TOPSIS approach to facilitate further development for the enhancement of traditional TOPSIS. The evaluation information of tenderers is presented as FNIFSs; thus the comparison rules of FNIFS are applied to determine the positive ideal tenderer (PIT) and negative ideal tenderer (NIT). Formulas for the distance calculation between two FNIFSs are utilized to compute the distance of each tenderer from the PIT and NIT.

## Overview of the Proposed Approach

**Fig 1:**
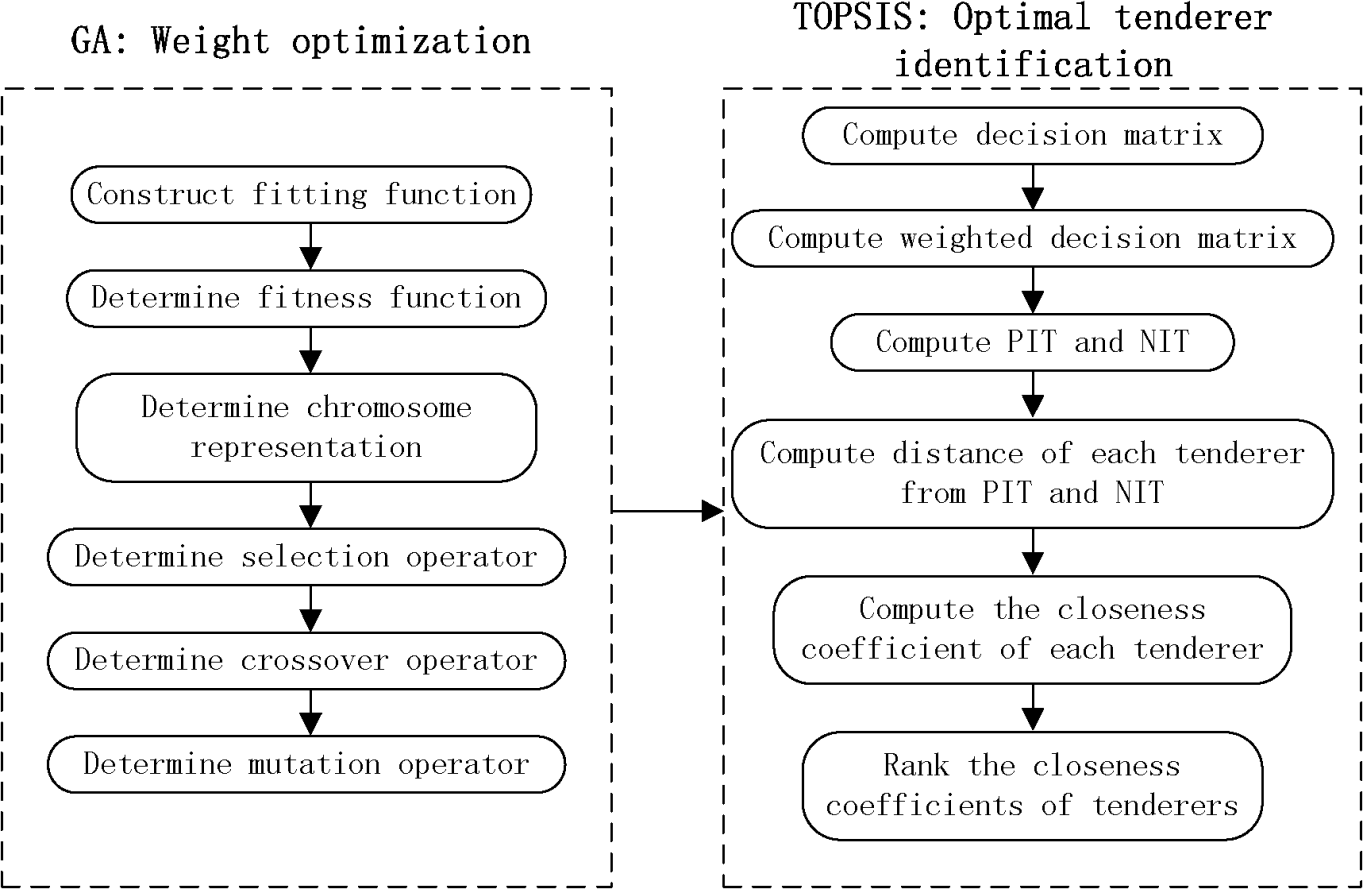
Detailed procedure of the proposed approach.

The proposed approach is a web-based bid evaluation of GeT to evaluate tenderers and identify the optimal tenderer efficiently and fairly under circumstance where the attributes of the tenderers are expressed as FNIFSs. This novel and hybrid approach contains two primary functions: weight optimization and optimal tenderer identification. Fig 1 illustrates the detailed procedure of the proposed approach.

In the process of weight optimization, a fitting function is constructed to express the limiting conditions that the optimal weights of the evaluation criteria must follow. Further, a modified GA approach is proposed to obtain the optimal weights of the evaluation criteria. This contains five parts: fitness function, chromosome representation, selection operator, crossover operator, and mutation operator.

In the optimal tenderer identifying stage, a modified FNIFS-based TOPSIS approach is proposed. This contains six principal steps that will be elaborated in section 5.

## Weight Optimization

Development and research of GA, especially studies of GA to resolve MADM problems, have grown exponentially since its introduction. Government E-tendering can be regarded as a MADM process manipulated by artificial intelligence. Considering that there may exist hundreds of millions of sets of weights of the evaluation criteria of tenderers (if the required precision has three decimal places), identifying the optimal set of weights becomes a complex and conflicting work; however, this can be easily obtained using GA [14]. Hu and Liao [25] proposed a GA-based learning method to obtain the degrees of importance of the evaluation criteria automatically. They employed this to identify the significant criteria for evaluating the electronic service quality of Internet banking. Inspired by this GA-based learning method, we propose the following weight optimization approach. In this stage, a fitting function will be conducted and a modified GA approach will be applied to determine the optimal weights of evaluation criteria of tenderers.

### A. Fitting function

Let *α*_*ijk*_ *= ‹(a*_*ijk*_,*b*_*ijk*_,*c*_*ijk)*_,*(l*_*ijk*_,*m*_*ijk*_,*p*_*ijk)*_ *› (i = 1*,*2*,*…*,*M*, *j = 1*,*2*,*…*,*N*, *k = 1*,*2*,*…*,*K)* be the fuzzy number intuitionistic fuzzy rating of expert *k* on the *j*th criterion of the *i*th tenderer and let *β*_*ik*_ *= ‹(a*_*ik*_,*b*_*ik*_,*c*_*ik)*_,*(l*_*ik*_,*m*_*ik*_,*p*_*ik)*_ *› (i = 1*,*2*,*…*,*M*, *k = 1*,*2*,*…*,*K)* be the fuzzy number intuitionistic fuzzy rating of expert *k* on the *i*th tenderer.

First, the aggregated average rating of all experts' ratings on one criterion of one tenderer must be calculated. Psychologically, the ratings of the experts will be significantly influenced by the subjectivity and prejudice of the experts, i.e., the membership and non-membership degrees of a fuzzy number intuitionistic fuzzy rating will influence each other. Thus, it is necessary to aggregate the membership and non-membership degrees of fuzzy number intuitionistic fuzzy ratings of all the experts separately, rather than aggregating them integrally, i.e., the operational rules presented in **Definition 2.3** are not suitable in this situation. Awasthi *et al*. [39] proposed an aggregation method, according to which the aggregated average fuzzy number intuitionistic fuzzy ratings will be:

$$\begin{array}{l} \alpha_{ij}=\langle \left(a_{ij},b_{ij},c_{ij}),(l_{ij},m_{ij},p_{ij}\right)\rangle \\ \;=\langle \left(\text{min}\{a_{ijk}\},\frac{1}{K}\sum_{k=1}^{K}b_{ijk},\text{max}\{c_{ijk}\}),(\text{min}\{l_{ijk}\},\frac{1}{K}\sum_{k=1}^{K}m_{ijk},\text{max}\{p_{ijk}\}\right)\rangle , \end{array}$$ (9)

$$\beta_{i}=\langle \left(\text{min}\{a_{ik}\},\frac{1}{K}\sum_{k=1}^{K}b_{ik},\text{max}\{c_{ik}\}),(\text{min}\{l_{ik}\},\frac{1}{K}\sum_{k=1}^{K}m_{ik},\text{max}\{p_{ik}\}\right)\rangle ,$$ (10)

where *k = 1*,*2*,*…*,*K*, *α*_*ij*_ is the aggregated fuzzy number intuitionistic fuzzy rating on the *j*th criterion of the *i*th tenderer and *β*_*i*_ is the aggregated overall rating on the *i*th tenderer. However, the above aggregation method has a fatal flaw when it is applied to FNIFS. The conditions:

$$\text{max}\{c_{ijk}\}+\text{max}\{p_{ijk}\}\leq 1,k=1,2,\ldots ,K,$$ (11)

$$\text{max}\{c_{ik}\}+\text{max}\{p_{ik}\}\leq 1,k=1,2,\ldots ,K,$$ (12)

cannot be proved and secured.

Therefore, we aggregate the fuzzy number intuitionistic fuzzy ratings of all the experts as follows:

$$\begin{array}{l} \alpha_{ij}=\langle \left(a_{ij},b_{ij},c_{ij}),(l_{ij},m_{ij},p_{ij}\right)\rangle \\ \;=\langle \left(\frac{1}{K}\sum_{k=1}^{K}a_{ijk},\frac{1}{K}\sum_{k=1}^{K}b_{ijk},\frac{1}{K}\sum_{k=1}^{K}c_{ijk}),(\frac{1}{K}\sum_{k=1}^{K}l_{ijk},\frac{1}{K}\sum_{k=1}^{K}m_{ijk},\frac{1}{K}\sum_{k=1}^{K}p_{ijk}\right)\rangle , \end{array}$$ (13)

$$\beta_{i}=\langle \left(\frac{1}{K}\sum_{k=1}^{K}a_{ik},\frac{1}{K}\sum_{k=1}^{K}b_{ik},\frac{1}{K}\sum_{k=1}^{K}c_{ik}),(\frac{1}{K}\sum_{k=1}^{K}l_{ik},\frac{1}{K}\sum_{k=1}^{K}m_{ik},\frac{1}{K}\sum_{k=1}^{K}p_{ik}\right)\rangle .$$ (14)

*β*_*i*_ can be regarded as the perceptive ratings rated by experts on the respective tenderers. Then, *β* ^*'*^_*i*_ denotes the real rating on the respective tenderers calculated as follows:

$$\beta_{i}^{'}=\sum_{j=1}^{N}\omega_{j}\alpha_{ij},$$ (15)

where *ω*_*j*_ is the weight of the *j*th criterion.

Based on the operation rules of FNIFS and (13), formula (15) can be transformed into:

$$\begin{array}{l} \beta_{i}^{'}=\langle \left(1-\prod_{j=1}^{N}{\left(1-a_{ij}\right)}^{\omega_{j}},1-\prod_{j=1}^{N}{\left(1-b_{ij}\right)}^{\omega_{j}},1-\prod_{j=1}^{N}{\left(1-c_{ij}\right)}^{\omega_{j}}),(\prod_{j=1}^{N}l_{ij}^{\omega_{j}},\prod_{j=1}^{N}m_{ij}^{\omega_{j}},\prod_{j=1}^{N}p_{ij}^{\omega_{j}}\right)\rangle \\ \;=\langle \left(1-\prod_{j=1}^{N}{\left(1-\frac{1}{K}\sum_{k=1}^{K}a_{ijk}\right)}^{\omega_{j}},1-\prod_{j=1}^{N}{\left(1-\frac{1}{K}\sum_{k=1}^{K}b_{ijk}\right)}^{\omega_{j}},1-\prod_{j=1}^{N}{\left(1-\frac{1}{K}\sum_{k=1}^{K}c_{ijk}\right)}^{\omega_{j}}),(\prod_{j=1}^{N}{\left(\frac{1}{K}\sum_{k=1}^{K}l_{ijk}\right)}^{\omega_{j}},\prod_{j=1}^{N}{\left(\frac{1}{K}\sum_{k=1}^{K}m_{ijk}\right)}^{\omega_{j}},\prod_{j=1}^{N}{\left(\frac{1}{K}\sum_{k=1}^{K}p_{ijk}\right)}^{\omega_{j}}\right)\rangle \end{array}$$ (16)

From (14) and (16), we can observe that the closer the values of *β*_*i*_ and *β* ^*'*^_*i*_, the better and more objective the corresponding weights of the evaluation criteria of tenderers. Consequently, we use the distance between FNIFSs to represent the closeness degree of two FNIFSs. Fan and Wang [40] defined a novel distance measure among FNIFSs, described as follows.

**Definition 4.1** Let *A = {α*_*11*_,*α*_*12*_,…,*α*_*1n*_ *}* and *B = {α*_*21*_,*α*_*22*_,…,*α*_*2n*_ *}* (*α*_*1i*_ *= ‹(a*_*1i*_,*b*_*1i*_,*c*_*1i*_ *)*,*(l*_*1i*_,*m*_*1i*_,*p*_*1i*_ *)›*, *α*_*2i*_ *= ‹(a*_*2i*_,*b*_*2i*_,*c*_*2i*_ *)*,*(l*_*2i*_,*m*_*2i*_,*p*_*2i*_ *)›*, *i = 1*,*2*,…,*n*) be two FNIFSs. *d(A*,*B)* denotes a three-dimensional distance measure of *A* and *B*, which can be represented as:

$$d(A,B)=\frac{\sum_{j=1}^{3}\lambda_{j}d^{j}(A,B)}{\sum_{j=1}^{3}\lambda_{j}}(\lambda_{j}\in [0,1]),$$ (17)

where *λ*_*j*_ is the weight of the distance measure *d* ^*j*^ *(A*,*B)*, *j = 1*,*2*,*3*,

$$d^{1}(A,B)=\frac{1}{n}\sum_{i=1}^{n}\left\{\frac{\left|a_{1i}-a_{2i}\right|+\left|l_{1i}-l_{2i}\right|}{4}+\frac{\text{max}(\left|a_{1i}-a_{2i}\right|,\left|l_{1i}-l_{2i}\right|)}{2}\right\},$$

$$d^{2}(A,B)=\frac{1}{n}\sum_{i=1}^{n}\left\{\frac{\left|b_{1i}-b_{2i}\right|+\left|m_{1i}-m_{2i}\right|}{4}+\frac{\text{max}(\left|b_{1i}-b_{2i}\right|,\left|m_{1i}-m_{2i}\right|)}{2}\right\},$$

$$d^{3}(A,B)=\frac{1}{n}\sum_{i=1}^{n}\left\{\frac{\left|c_{1i}-c_{2i}\right|+\left|p_{1i}-p_{2i}\right|}{4}+\frac{\text{max}(\left|c_{1i}-c_{2i}\right|,\left|p_{1i}-p_{2i}\right|)}{2}\right\}.$$

Therefore, according to **Definition 4.1**, (14), and (16), the distance between *β = {β*_*1*_,*β*_*2*_,…,*β*_*M*_ *}* and *β* ^*'*^ *= {β* ^*'*^_*1*_,*β* ^*'*^_*2*_,*…*,*β* ^*'*^_*M*_ *}* can be represented as:

$$d(\beta ,\beta^{'})=\left\{\frac{\lambda_{1}}{M}\sum_{i=1}^{M}\left[\frac{\Delta_{1}+\Delta_{2}}{4}+\frac{\text{max}(\Delta_{1},\Delta_{2})}{2}\right]+\frac{\lambda_{2}}{M}\sum_{i=1}^{M}\left[\frac{\Delta_{3}+\Delta_{4}}{4}+\frac{\text{max}(\Delta_{3},\Delta_{4})}{2}\right]+\frac{\lambda_{3}}{M}\sum_{i=1}^{M}\left[\frac{\Delta_{5}+\Delta_{6}}{4}+\frac{\text{max}(\Delta_{5},\Delta_{6})}{2}\right]\right\}/\sum_{j=1}^{3}\lambda_{j}$$ (18)

$$\text{Where}\;\Delta_{1}=\left|1-\prod_{j=1}^{N}{\left(1-\frac{1}{K}\sum_{k=1}^{K}a_{ijk}\right)}^{\omega_{j}}-\frac{1}{K}\sum_{k=1}^{K}a_{ik}\right|,\;\Delta_{2}=\left|\prod_{j=1}^{N}{\left(\frac{1}{K}\sum_{k=1}^{K}l_{ijk}\right)}^{\omega_{j}}-\frac{1}{K}\sum_{k=1}^{K}l_{ik}\right|,$$

$$\Delta_{3}=\left|1-\prod_{j=1}^{N}{\left(1-\frac{1}{K}\sum_{k=1}^{K}b_{ijk}\right)}^{\omega_{j}}-\frac{1}{K}\sum_{k=1}^{K}b_{ik}\right|,\;\Delta_{4}=\left|\prod_{j=1}^{N}{\left(\frac{1}{K}\sum_{k=1}^{K}m_{ijk}\right)}^{\omega_{j}}-\frac{1}{K}\sum_{k=1}^{K}m_{ik}\right|,$$

$$\Delta_{5}=\left|1-\prod_{j=1}^{N}{\left(1-\frac{1}{K}\sum_{k=1}^{K}c_{ijk}\right)}^{\omega_{j}}-\frac{1}{K}\sum_{k=1}^{K}c_{ik}\right|,\;\text{and}\;\Delta_{6}=\left|\prod_{j=1}^{N}{\left(\frac{1}{K}\sum_{k=1}^{K}p_{ijk}\right)}^{\omega_{j}}-\frac{1}{K}\sum_{k=1}^{K}p_{ik}\right|.$$

Finally, the fitting function will be presented as:

$$\left\{ \begin{array}{l} \text{min}\;d(\beta ,\beta^{'})\\ \sum_{j=1}^{N}\omega_{j}=1 \end{array}\right.,$$ (19)

That is, if a set of weights *ω = {ω*_*1*_,*ω*_*2*_,*…*,*ω*_*N*_ *}* satisfies the above formula, i.e., minimizes the distance between *β* and *β* ^*'*^, *ω* can be regarded as the optimal set of weights of the evaluation criteria of the tenderers.

### B. Fitness function

In the GA process, there is a positive value called *fitness value* that is used to reflect the degree of “goodness” of a chromosome. The fittest individual is the one with the greatest *fitness value*. Based on the description in the previous section, the fitness function will be represented as:

$$f_{\omega }=\frac{1}{1+d(\beta ,\beta^{'})},$$ (20)

where *f*_*ω*_ denotes the fitness value of the weight set *ω*. *f*_*ω*_ will be updated with every new generation of the GA. Therefore, the purpose of the GA is to determine the weight set with the greatest fitness value by minimizing *d(β*,*β* ^*'*^ *)*.

### C. Chromosome representation

The GA algorithm presumes that each potential solution of a problem can be regarded as a chromosome. This is because each potential solution is comprised of a set of parameters in the same manner that each chromosome is composed of a number of genes. In the GA approach to a problem, it is important to determine the adequate chromosome representation of the problem [27]. For the problem studied in this paper, we adopt the real-value representation scheme because floating-point representation is computationally faster and more consistent than the run-to-run basis [41]. For each weight parameter, the required precision has three decimal places. For example, if there are six evaluation criteria, one randomly generated chromosome may be “0.121–0.332–0.053–0.268–0.105–0.121”.

### D. Selection operator

A proficient selection mechanism is required to select suitable parents to reproduce effective offspring. To generate new candidate parents efficiently, we adopt a ranking scheme [42] that has proven to be effective in the prevention of premature convergence and to accelerate the search when the population approaches convergence [43]. During the ranking scheme process, chromosomes are compared to define their rank and determine the chromosomes to be selected as parents.

In this paper, an initial population *N*_*pp*_ of individuals is generated randomly as candidate parents for the crossover and mutation operators. After the crossover and mutation operations, *N*_*cp*_ daughter chromosomes will exist. Among these *N*_*cp*_ ranked daughter chromosomes, only the top *N*_*pp*_ generations will be selected as parents for reproducing the next generations.

### E. Crossover operator

**Fig 2:**
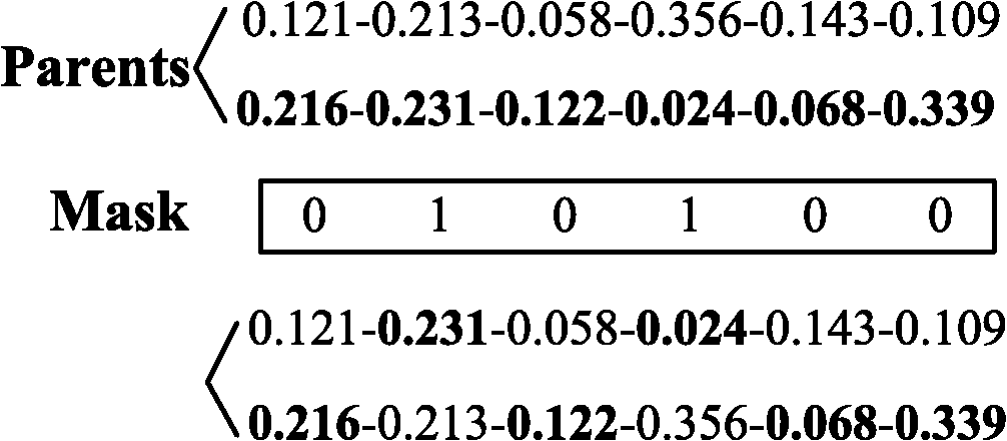
Example of uniform crossover.

Although a one-point crossover mechanism is a replication of the biological process, it has drawbacks when addressing real-value-represented chromosomes. Therefore, we adopt a uniform crossover that generates offspring based on a randomly generated crossover mask. The uniform crossover exchanges bits rather than segments and can combine features regardless of their relative locations [14]. This makes uniform crossover a superior operator for real-value-represented chromosomes. The operation is displayed in Fig 2.

From Fig 2, we can observe that the new resultant offspring contains genes from both parents. The number of effective crossover points is not fixed; it is the average *L/2* (*L* is the length of chromosome) [14].

Usually, the sum of the weight values of each offspring will not equal one. This means the new resultant offspring is a “bad” generation. For those “bad” generations where the sum of all the weight values is less than one, our action is to change the value of one randomly selected gene, that is not a crossover point, to force the sum of the values of all the genes to equal one. For example, for the upper offspring in Fig 2, “0.121–0.231–0.058–0.024–0.143–0.109”, we select the first gene “0.121” randomly and change its value to “0.435”. Thus, the sum of all the weight values will be equal to one. The “bad” generations whose sum of all the weight values are greater than one are eliminated.

### F. Mutation operator

**Fig 3:**
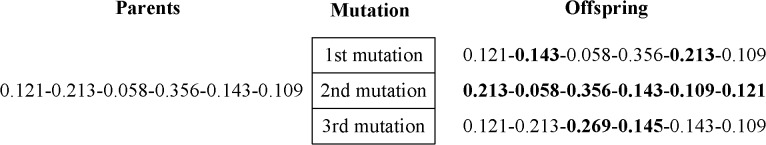
Example of mutation operation.

The process of mutation is applied to a single offspring after the crossover exercise. Inspired from our previous work [44], we propose a new mutation operator to apply to a chromosome three times to obtain the greatest number of possible variations. The following steps, with an illustrative example in Fig 3, demonstrate this new mutation operator:

1. Choose one chromosome from the offspring generated by the previous crossover operation.
2. In the first mutation, two randomly chosen genes in the selected chromosome swap; the remaining genes remain the same. In Fig 3, the second gene “0.143” is swapped with the fifth gene “0.213”. Because the values and positions of the remaining genes are the same, the mutated offspring is represented as “0.121–0.143–0.058–0.356–0.213–0.109”.
3. In the second mutation, each gene of the selected chromosome swaps with the following, one by one from left to right successively to the last gene. In Fig 3, the new generated chromosome is “0.213–0.058–0.356–0.143–0.109–0.121”.
4. In the third mutation, two randomly selected gene values shift while maintaining the sum of the gene values equal to one. In Fig 3, the values of the third and fourth genes have been modified and the new resultant offspring is “0.121–0.213–0.269–0.145–0.143–0.109”.
5. If any of the mutated chromosomes are the same, the repeated chromosomes are removed.

**Fig 4:**
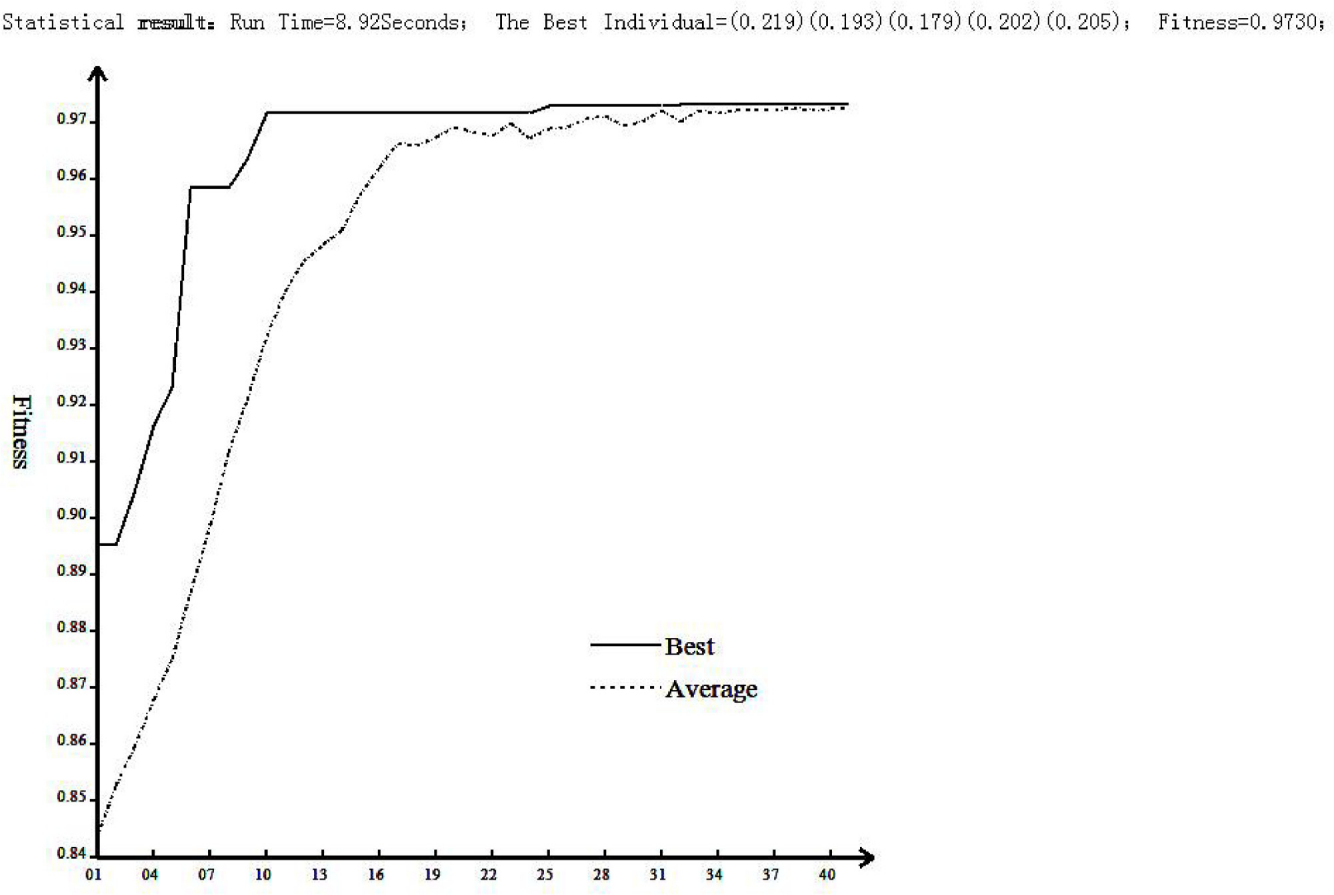
Computational results of the proposed GA-based weight optimization method.

Fig 4 presents the computational results of the proposed GA-based weight optimization method. The experiment was developed in the C# programming language. We can determine from the figure that the fitness value finally converged to approximately 0.97. Actually, based on the calculated results, the converged fitness value was 0.9730 and the corresponding optimal weight vector was {0.219, 0.193, 0.179, 0.202, 0.205}, i.e., the weights of the evaluation criteria “safety”, “function”, “artistry”, “feasibility”, and “price” are 0.219, 0.193, 0.179, 0.202, and 0.205, respectively. The weight of “safety” is the greatest; the weight of “artistry” is the least. The weights of “feasibility” and “price” are similar and are slightly greater than “function”. This result fits the actual situation reasonably well and confirms the effectiveness of the GA-based weight optimization method.

## Optimal Tenderer Identification

The fuzzy TOPSIS approach involves fuzzy assessments of the criteria and alternatives in TOPSIS [33]. In this paper, the assessments of the evaluation criteria are expressed as FNIFSs. Therefore, we combine TOPSIS with FNIFS and propose a modified FNIFS-based TOPSIS approach. The steps of this modified TOPSIS are presented following.

(1) Compute the decision matrix. According to part A of Section 4, the decision matrix of the tenderers (*D*_*ij*_) is constructed as:

$$D_{ij}=\left[\begin{array}{cccc} \alpha_{11} & \alpha_{12} & \ldots & \alpha_{1N}\\ \alpha_{21} & \alpha_{22} & \ldots & \alpha_{2N}\\ \ldots & \ldots & \ldots & \ldots \\ \alpha_{M1} & \alpha_{M2} & \ldots & \alpha_{MN} \end{array}\right],\;i=1,2,\ldots ,M,j=1,2,\ldots ,N.$$ (21)

(2) Compute the weighted decision matrix. The weighted decision matrix of the tenderers (*R*_*ij*_) is constructed as: follows:

$$R_{ij}={\left[r_{ij}\right]}_{M\times N},\;i=1,2,\ldots ,M,j=1,2,\ldots ,N,$$ (22)

where:

$$\begin{array}{l} r_{ij}=\omega_{j}\times \alpha_{ij}\\ \;=\langle \left(1-{\left(1-\frac{1}{K}\sum_{k=1}^{K}a_{ijk}\right)}^{\omega_{j}},1-{\left(1-\frac{1}{K}\sum_{k=1}^{K}b_{ijk}\right)}^{\omega_{j}},1-{\left(1-\frac{1}{K}\sum_{k=1}^{K}c_{ijk}\right)}^{\omega_{j}}),({\left(\frac{1}{K}\sum_{k=1}^{K}l_{ijk}\right)}^{\omega_{j}},{\left(\frac{1}{K}\sum_{k=1}^{K}m_{ijk}\right)}^{\omega_{j}},{\left(\frac{1}{K}\sum_{k=1}^{K}p_{ijk}\right)}^{\omega_{j}}\right)\rangle \end{array}$$ (23)

(3) Compute PIT and NIT. The positive ideal solution is composed of the best assessment values; the negative ideal solution is composed of the worst assessment values. According to the philosophy of the TOPSIS approach, the alternative that is the closest to the positive ideal solution and the farthest from the negative ideal solution is selected as the optimal alternative. Let *T*_*PIT*_ be the FNIFS of PIT and *T*_*NIT*_ be the FNIFS of NIT, then *T*_*PIT*_ and *T*_*NIT*_ can be computed as follows:

$$T_{PIT}=\left\{\text{max}\{r_{i1}\},\text{max}\{r_{i2}\},\ldots ,\text{max}\{r_{iN}\}\right\},i=1,2,\ldots ,M,$$ (24)

$$T_{NIT}=\left\{\text{min}\{r_{i1}\},\text{min}\{r_{i2}\},\ldots ,\text{min}\{r_{iN}\}\right\},i=1,2,\ldots ,M.$$ (25)

According to **Definition 2.6** and (23), the score and accuracy functions of *r*_*ij*_ can be represented as:

$$\begin{array}{r} S^{*}(r_{ij})=\frac{4-{\left(1-\frac{1}{K}\sum_{k=1}^{K}a_{ijk}\right)}^{\omega_{j}}-2{\left(1-\frac{1}{K}\sum_{k=1}^{K}b_{ijk}\right)}^{\omega_{j}}-{\left(1-\frac{1}{K}\sum_{k=1}^{K}c_{ijk}\right)}^{\omega_{j}}-{\left(\frac{1}{K}\sum_{k=1}^{K}l_{ijk}\right)}^{\omega_{j}}-2{\left(\frac{1}{K}\sum_{k=1}^{K}m_{ijk}\right)}^{\omega_{j}}-{\left(\frac{1}{K}\sum_{k=1}^{K}p_{ijk}\right)}^{\omega_{j}}}{4}\\ i=1,2,\ldots ,M,j=1,2,\ldots ,N \end{array}\;,$$ (26)

$$\begin{array}{r} H^{*}(r_{ij})=\frac{4-{\left(1-\frac{1}{K}\sum_{k=1}^{K}a_{ijk}\right)}^{\omega_{j}}-2{\left(1-\frac{1}{K}\sum_{k=1}^{K}b_{ijk}\right)}^{\omega_{j}}-{\left(1-\frac{1}{K}\sum_{k=1}^{K}c_{ijk}\right)}^{\omega_{j}}+{\left(\frac{1}{K}\sum_{k=1}^{K}l_{ijk}\right)}^{\omega_{j}}+2{\left(\frac{1}{K}\sum_{k=1}^{K}m_{ijk}\right)}^{\omega_{j}}+{\left(\frac{1}{K}\sum_{k=1}^{K}p_{ijk}\right)}^{\omega_{j}}}{4}\\ i=1,2,\ldots ,M,j=1,2,\ldots ,N \end{array}\;,$$ (27)

Then, according to the comparison rules in **Definition 2.6**, *T*_*PIT*_ and *T*_*NIT*_ can be obtained.

(4) Compute the distance of each tenderer from PIT and NIT. Let *T*_*i*_ *= {r*_*i1*_,*r*_*i2*_,…,*r*_*iN*_ *}* be the FNIFS of the *i*th tenderer and let *max{r*_*ij*_ *} = r* ^*+*^_*ij*_ and *min{r*_*ij*_ *} = r* ^*-*^_*ij*_, then *T*_*PIT*_ *= {r* ^*+*^_*i1*_,*r* ^*+*^_*i2*_,…,*r* ^*+*^_*iN*_ *} (r* ^*+*^_*ij*_ *= <(a* ^*+*^_*ij*_,*b* ^*+*^_*ij*_,*c* ^*+*^_*ij*_ *)*,*(l* ^*+*^_*ij*_,*m* ^*+*^_*ij*_,*p* ^*+*^_*ij*_ *)>*, *j = 1*,*2*,…,*N)* and *T*_*NIT*_ *= {r* ^*-*^_*i1*_,*r* ^*-*^_*i2*_,…,*r* ^*-*^_*iN*_ *} (r* ^*-*^_*ij*_ *= <(a* ^*-*^_*ij*_,*b* ^*-*^_*ij*_,*c* ^*-*^_*ij*_ *)*,*(l* ^*-*^_*ij*_,*m* ^*-*^_*ij*_,*p* ^*-*^_*ij*_ *)>*, *j = 1*,*2*,…,*N)*. According to **Definition 4.1** and (23), we can calculate the distance of each alternatives from PIT and NIT as follows:

$$d^{+}(T_{i},T_{PIT})=\left\{\frac{\lambda_{1}}{N}\sum_{j=1}^{N}\left[\frac{\Delta_{1}^{+}+\Delta_{2}^{+}}{4}+\frac{\text{max}(\Delta_{1}^{+},\Delta_{2}^{+})}{2}\right]+\frac{\lambda_{2}}{N}\sum_{j=1}^{N}\left[\frac{\Delta_{3}^{+}+\Delta_{4}^{+}}{4}+\frac{\text{max}(\Delta_{3}^{+},\Delta_{4}^{+})}{2}\right]+\frac{\lambda_{3}}{N}\sum_{j=1}^{N}\left[\frac{\Delta_{5}^{+}+\Delta_{6}^{+}}{4}+\frac{\text{max}(\Delta_{5}^{+},\Delta_{6}^{+})}{2}\right]\right\}/\sum_{i=1}^{3}\lambda_{i},$$ (28)

where *d* ^*+*^ *(T*_*i*_,*T*_*PIT*_ *)* is the distance of *T*_*i*_ from *T*_*PIT*_, $\Delta_{1}^{+}=\left|1-{\left(1-\frac{1}{K}\sum_{k=1}^{K}a_{ijk}\right)}^{\omega_{j}}-a_{ij}^{+}\right|$, $\Delta_{2}^{+}=\left|{\left(\frac{1}{K}\sum_{k=1}^{K}l_{ijk}\right)}^{\omega_{j}}-l_{ij}^{+}\right|$, $\Delta_{3}^{+}=\left|1-{\left(1-\frac{1}{K}\sum_{k=1}^{K}b_{ijk}\right)}^{\omega_{j}}-b_{ij}^{+}\right|$, $\Delta_{4}^{+}=\left|{\left(\frac{1}{K}\sum_{k=1}^{K}m_{ijk}\right)}^{\omega_{j}}-m_{ij}^{+}\right|$, $\Delta_{5}^{+}=\left|1-{\left(1-\frac{1}{K}\sum_{k=1}^{K}c_{ijk}\right)}^{\omega_{j}}-c_{ij}^{+}\right|$, $\Delta_{6}^{+}=\left|{\left(\frac{1}{K}\sum_{k=1}^{K}p_{ijk}\right)}^{\omega_{j}}-p_{ij}^{+}\right|$.

$$d^{-}(T_{i},T_{NIT})=\left\{\frac{\lambda_{1}}{N}\sum_{j=1}^{N}\left[\frac{\Delta_{1}^{-}+\Delta_{2}^{-}}{4}+\frac{\text{max}(\Delta_{1}^{-},\Delta_{2}^{-})}{2}\right]+\frac{\lambda_{2}}{N}\sum_{j=1}^{N}\left[\frac{\Delta_{3}^{-}+\Delta_{4}^{-}}{4}+\frac{\text{max}(\Delta_{3}^{-},\Delta_{4}^{-})}{2}\right]+\frac{\lambda_{3}}{N}\sum_{j=1}^{N}\left[\frac{\Delta_{5}^{-}+\Delta_{6}^{-}}{4}+\frac{\text{max}(\Delta_{5}^{-},\Delta_{6}^{-})}{2}\right]\right\}/\sum_{i=1}^{3}\lambda_{i}$$ (29)

where *d* ^*-*^ *(T*_*i*_,*T*_*NIT*_ *)* is the distance of *T*_*i*_ from *T*_*NIT*_, $\Delta_{1}^{-}=\left|1-{\left(1-\frac{1}{K}\sum_{k=1}^{K}a_{ijk}\right)}^{\omega_{j}}-a_{ij}^{-}\right|$, $\Delta_{2}^{-}=\left|{\left(\frac{1}{K}\sum_{k=1}^{K}l_{ijk}\right)}^{\omega_{j}}-l_{ij}^{-}\right|$, $\Delta_{3}^{-}=\left|1-{\left(1-\frac{1}{K}\sum_{k=1}^{K}b_{ijk}\right)}^{\omega_{j}}-b_{ij}^{-}\right|$, $\Delta_{4}^{-}=\left|{\left(\frac{1}{K}\sum_{k=1}^{K}m_{ijk}\right)}^{\omega_{j}}-m_{ij}^{-}\right|$, $\Delta_{5}^{-}=\left|1-{\left(1-\frac{1}{K}\sum_{k=1}^{K}c_{ijk}\right)}^{\omega_{j}}-c_{ij}^{-}\right|$, $\Delta_{6}^{-}=\left|{\left(\frac{1}{K}\sum_{k=1}^{K}p_{ijk}\right)}^{\omega_{j}}-p_{ij}^{-}\right|$.

(5) Compute the closeness coefficient of each tenderer. The closeness coefficient (*CO*_*i*_) represents the optimization degree of the *i*th tenderer and is represented as:

$$CO_{i}=\frac{d^{-}(T_{i},T_{NIT})}{d^{+}(T_{i},T_{PIT})+d^{-}(T_{i},T_{NIT})},i=1,2,\ldots ,M.$$ (30)

(6) Rank the closeness coefficients. The closeness coefficients of all the tenderers are ranked in decreasing order. The tenderer with the greatest closeness coefficient is regarded as the optimal tenderer.

## Illustrative Example of the Prototype System

In this section, we present an example of a GeT system searching for the optimal tenderer to test the practicality and effectiveness of the proposed approach. The software prototype was developed in the Java programming language and ExtJs framework. The development and operating environment included the Windows 7 operating system, MyEclipse 10 compiler software with Java EE 6.0, Tomcat 7.0 server, and Java virtual machine (JVM). The source code of the software prototype was given as supporting information file (S1 Code), and some related Jar files were also given (S1 File, S2 File, S3 File and S4 File). Furthermore, an introduction file about the implementation of the source code was given (S2 Text).

**Fig 5:**
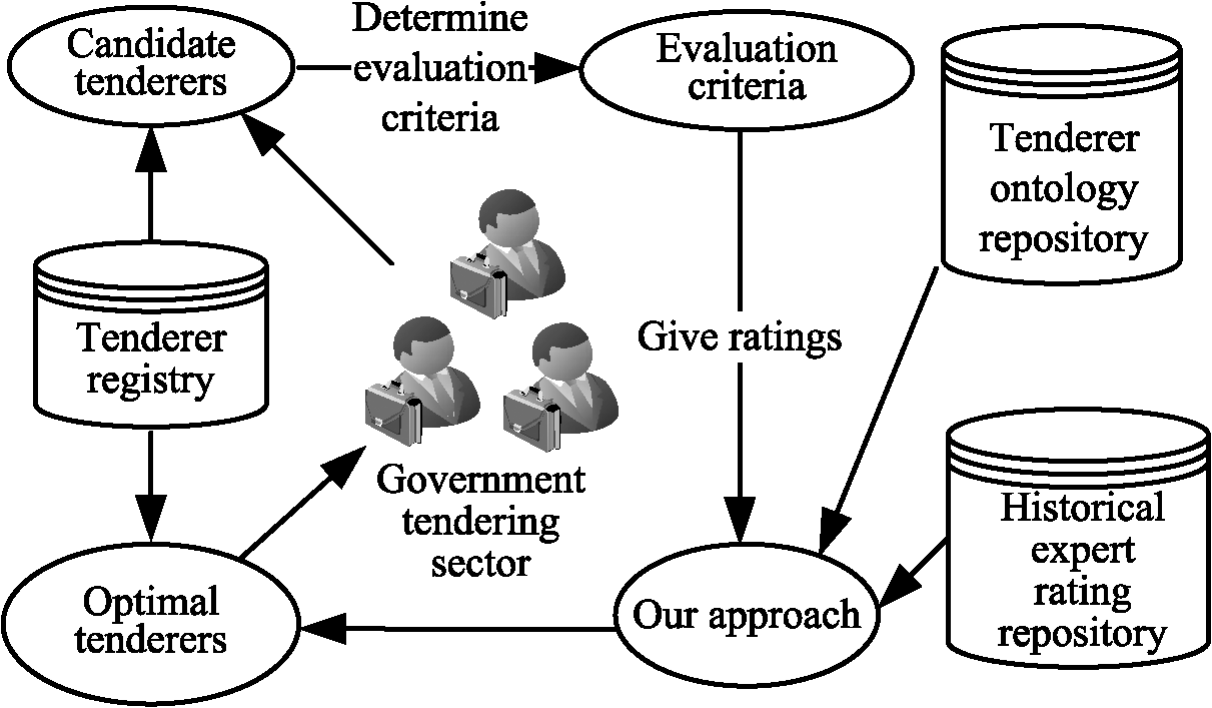
Operational procedure of finding the optimal tenderer.

The purpose of the example is to demonstrate the search for the tenderer with the greatest closeness coefficient value in a specified context. Fig 5 illustrates the operational procedure of determining the optimal tenderer using the proposed approach. To begin, several candidate tenderers are screened from all the effective tenderers that are saved in the tenderer registry. The evaluation criteria are selected or input. The evaluation experts provide their ratings on each criterion of the tenderers and their overall ratings on each tenderer. Then, the proposed approach infers the tenderer with the greatest closeness coefficient value from all the candidate tenderers. The historical expert ratings and tenderer information are extracted from a historical expert rating repository and tenderer ontology repository, respectively. Our previous researches [45–46] have developed a rich body of OWL-based (OWL, ontology Web language) service ontologies that can provide valid reference for the current approach.

**Fig 6:**
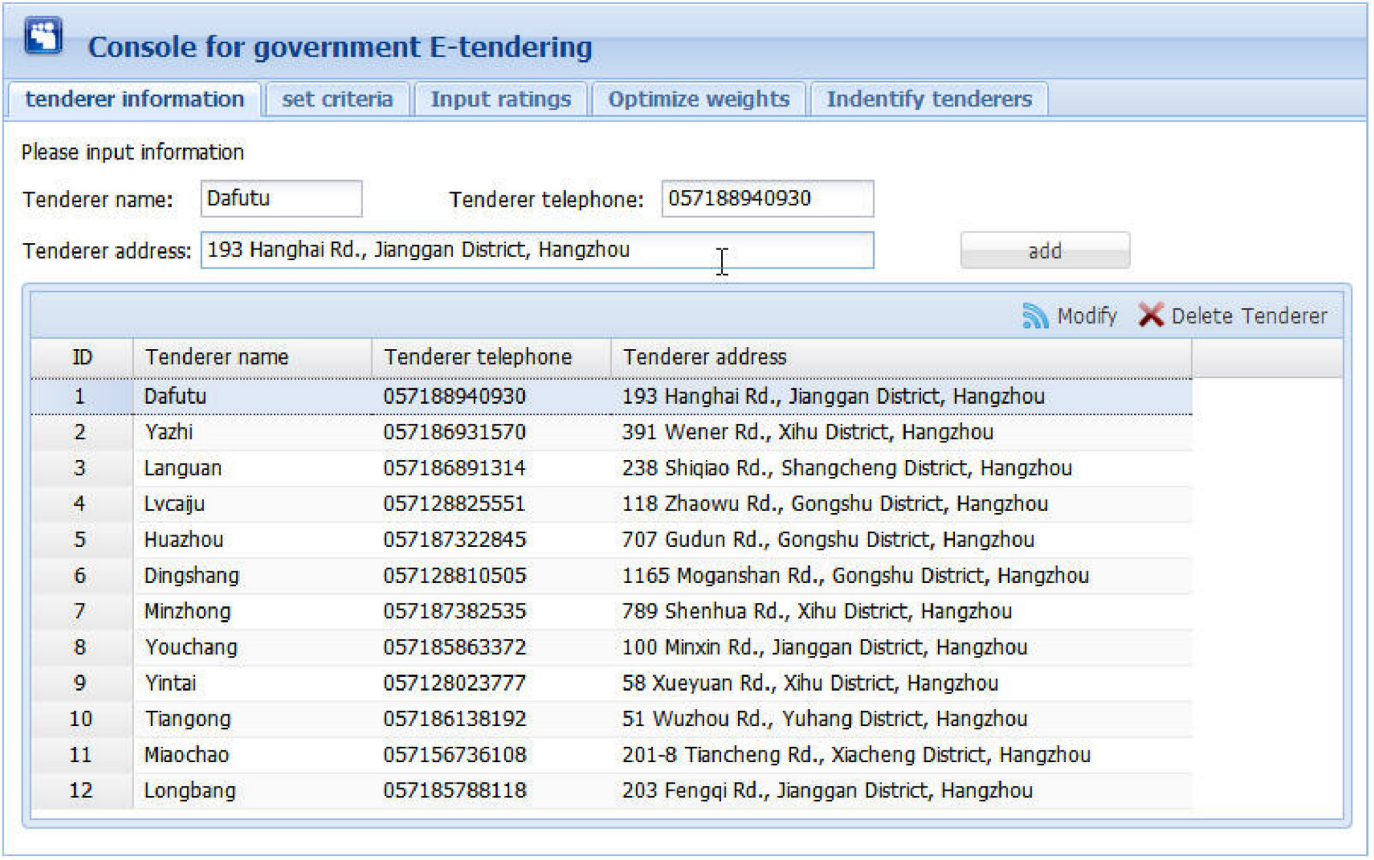
Graphic interface for inputting tenderer information.

**Fig 7:**
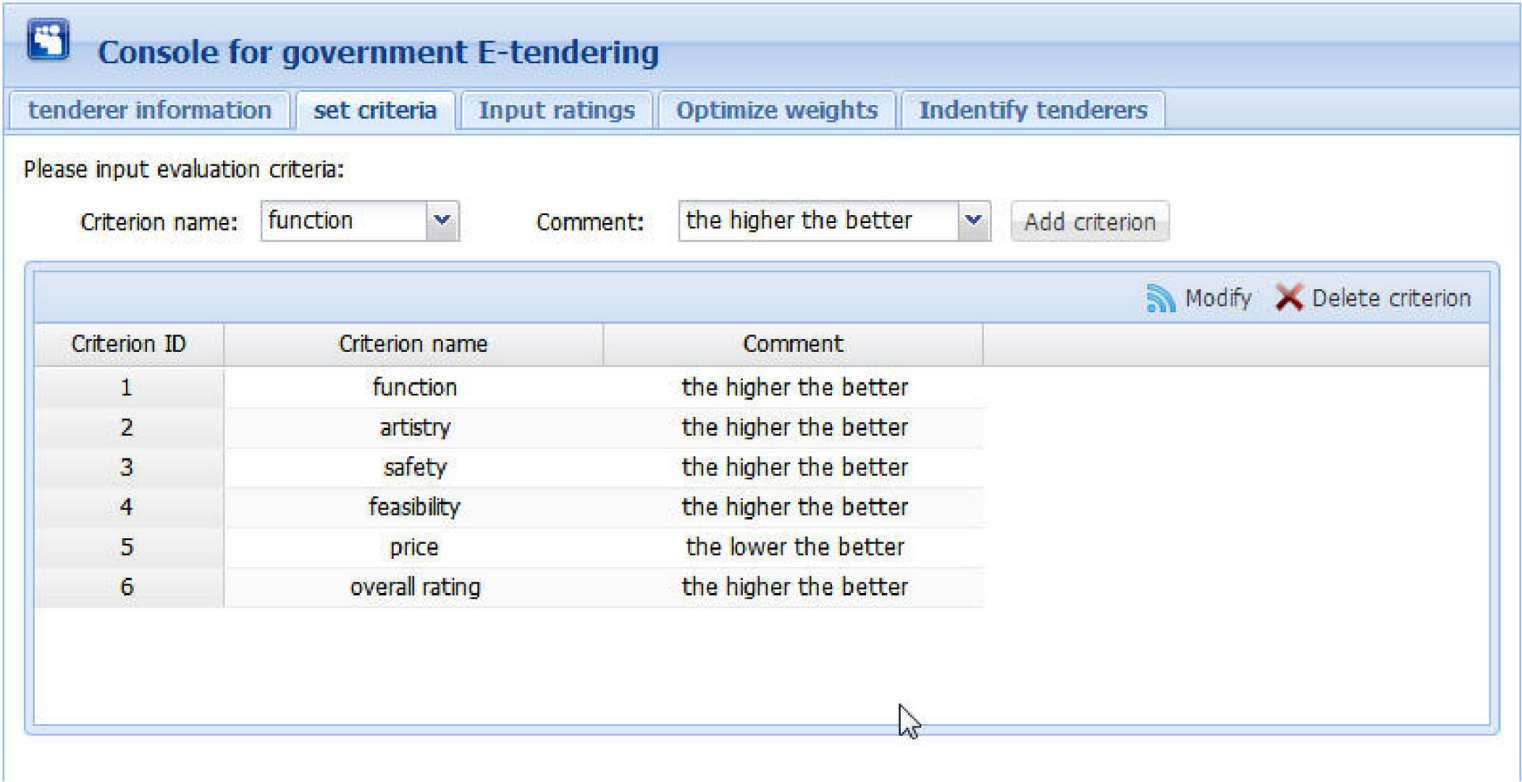
Graphical interface for setting evaluation criteria.

**Fig 8:**
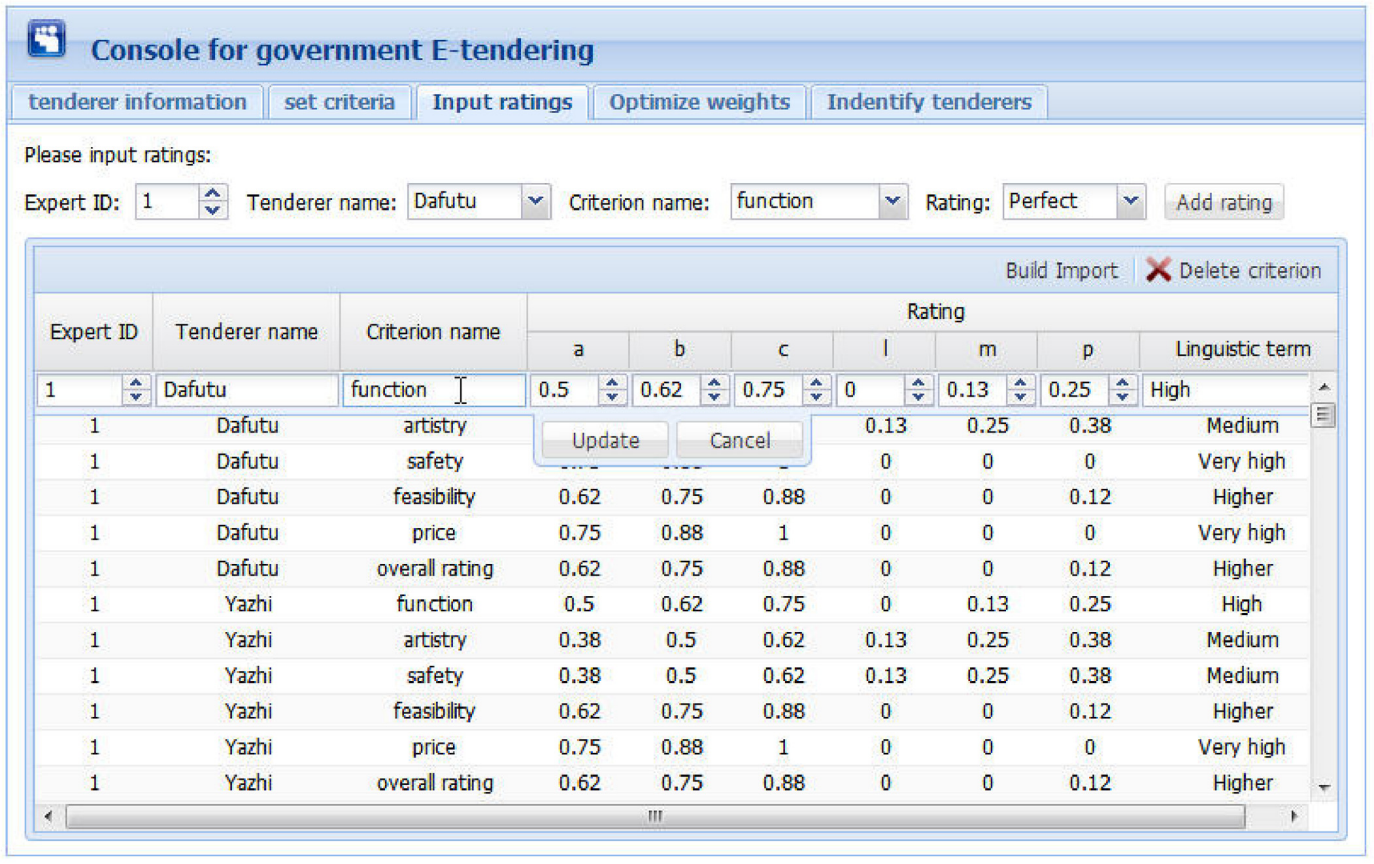
Graphical interface for inputting ratings of experts.

**Fig 9:**
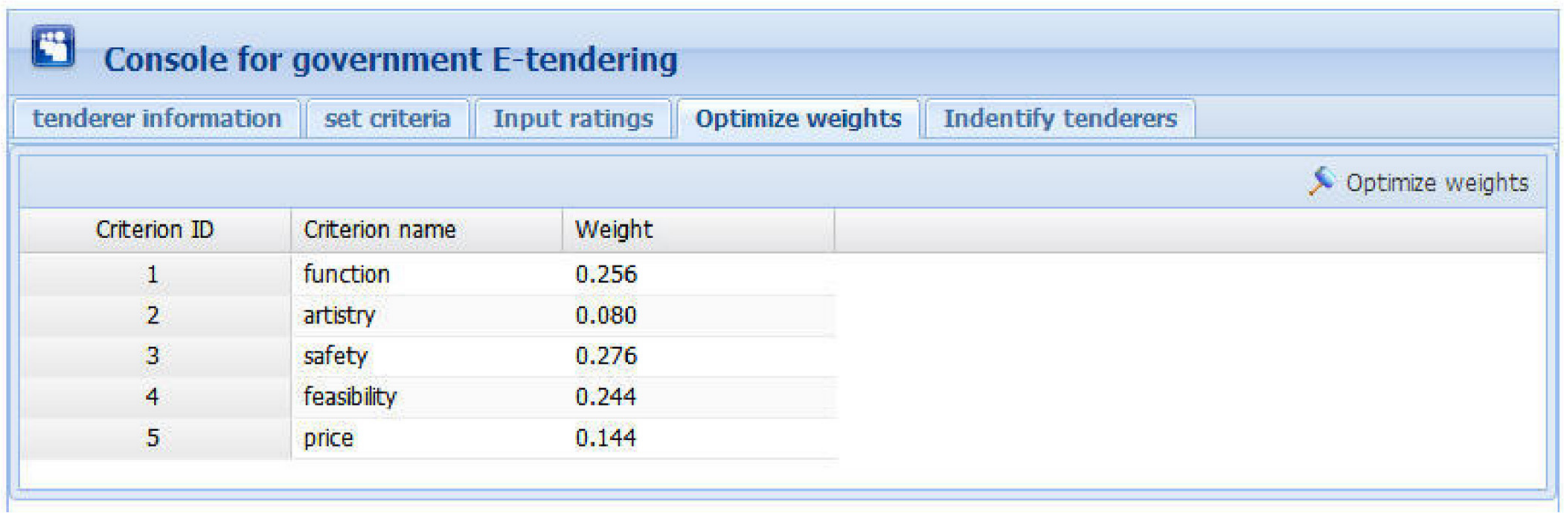
Graphical interface for optimizing weights.

**Fig 10:**
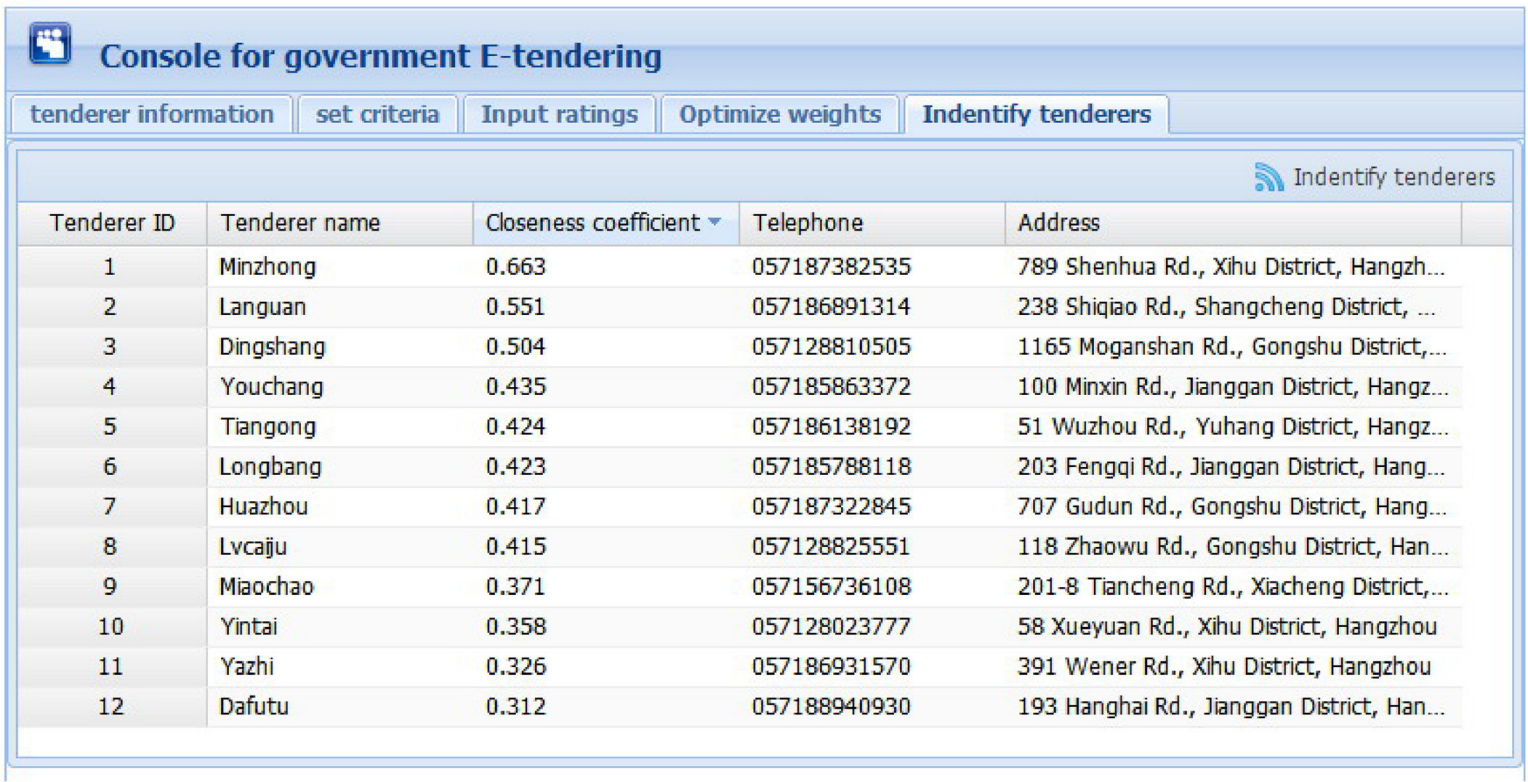
Graphical interface for identifying tenderers.

Figs 6 to 10 present the graphical interfaces for optimal tenderer identification in the prototype system. The process of identifying the optimal tenderer is as follows:

1. We assume that a governmental department wants to redecorate an entire office block and the government-tendering sector wishes to locate an appropriate decoration firm through open tendering online. During this online open tendering, 12 tenderers have submitted their bidding documents. They are “Dafutu”, “Yazhi”, “Languan”, “Lvcaiju”, “Huazhou”, “Dingshang”, “Minzhong”, “Youchang”, “Yintai”, “Tiangong”, “Miaochao” and “Longbang”. The detailed tenderer information was given as supporting information file (S1 Text). To begin, the government tendering sector must input basic information for these tenderers (see Fig 6), including tenderer name, telephone, and address, into the prototype system. The input information can be modified or deleted, if necessary.
2. After inputting the basic tenderer information, the government-tendering sector must define the evaluation criteria (see top of Fig 7) including the criterion name and comment, e.g., the lower the better or the higher the better. Then, clicking the “Add criterion” button presents a set of evaluation criteria as indicated in the Fig 7 window. In this example, there are five evaluation criteria including “function”, “artistry”, “safety”, “feasibility”, and “price”. The “overall rating” criterion represents the overall ratings of experts on the 12 candidate tenderers. The input information can be modified or deleted, if necessary.
3. Upon setting the evaluation criteria, the ratings of 5 experts on the 5 evaluation criteria and 12 candidate tenderers must be input, including expert ID, tenderer name, criterion name, and corresponding rating. During this process, the ratings are expressed in vague language. Herrera and Martinez [47] illustrated a seven-term linguistic term set and used a triangular fuzzy number as a linguistic descriptor. Inspired from this, we propose a nine-term linguistic term set and apply FNIFS as the linguistic descriptor, presented as “Perfect = <(0.88,1,1),(0,0,0)>”, “Very high = <(0.75,0.88,1),(0,0,0)>”, “Higher = <(0.62,0.75,0.88),(0,0,0.12)>”, “High = <(0.50,0.62,0.75),(0,0.13,0.25)>”, “Medium = <(0.38,0.50,0.62),(0.13,0.25,0.38)>”, “Low = <(0.26,0.38,0.50),(0.25,0.38,0.50)>”, “Lower = <(0.13,0.26,0.38),(0.38,0.50,0.62)>”, “Very Low = <(0,0.13,0.26),(0.50,0.62,0.74)>”, and “None = <(0,0,0.13),(0.62,0.74,0.87)>”. If a file exists with evaluation information in “txt” format, the users can simply import the file. In this example, the evaluation information was given as supporting information file (S1 Data). Alternatively, users can manually enter the evaluation information. An inexperienced user can select ratings that are expressed as vague language. Then, by clicking the “Add rating” button, the rating of one corresponding criterion of one tenderer rated by one expert will be presented as the corresponding FNIFS in the window of Fig 8. The first line “1 Dafutu safety a = 0.5 b = 0.62 c = 0.75 l = 0.00 m = 0.13 p = 0.25 (High)” means the FNIFS expression of the rating provided by “expert 1” for the criterion “safety” of tenderer “Dafutu” is <(0.5,0.62,0.75),(0,0.13,0.25)> and its corresponding vague language is “High”. An experienced user can edit the ratings to make them more practical for the specified context by double clicking the corresponding row. Users can also delete ratings by clicking the “Delete rating” button.
4. After inputting all the information, by clicking the “Optimize weights” button in the top right corner of the window displayed in Fig 9, one can obtain the optimal weights of the five evaluation criteria. Because of the nature of GA, the weight value of each criterion is not invariable, i.e., each calculation makes the weight value of each criterion get tiny change. However, these changes are reasonable and acceptable. In this example, the optimal weight values are shown in Fig 9, indicated as “function 0.256”, “artistry 0.080”, “safety 0.276”, “feasibility 0.244”, and “price 0.144”. We can observe that compared with “artistry”, the remaining four criteria are more critical to the government in selecting a suitable decoration firm. This is realistic.
5. Finally, by clicking the “Identify tenderers” button in the top right corner of the window of Fig 10, we can obtain the ranking list of the 12 candidate tenderers with their corresponding closeness coefficient value in decreasing order, including telephone number and address. Because of the tiny variability of weight values, the ranking of all candidate tenderers is also not invariable, i.e., the relative ranking of tenderers with almost the same closeness coefficient values may vary as the corresponding weight values change. Nonetheless, the optimal tenderer still can be recognized. It is just a matter of the number of the optimal tenderer, i.e., there may be exist several optimal tenderers. In this example, the decoration firm “Minzhong” has the greatest closeness coefficient value, 0.663, meaning this firm is the optimal tenderer from the government search. That is, “Minzhong” is the most suitable tenderer for the government requirements among the 12 candidate tenderers.

## Conclusion

In this paper, we proposed a hybrid approach combining GA and TOPSIS for government E-tendering to identify the optimal tenderer efficiently and fairly under circumstances where the attributes of the tenderers were expressed as FNIFSs. The main contributions of this paper are summarized as:

- Development of a methodology for government E-tendering using intuitionistic fuzzy information that can calculate vague language facilitating the task of evaluating the tenderers more realistically and objectively.
- Development of a government E-tendering system that exploits the superiorities of tendering to the utmost, enabling improved transparency and reduced cost.
- Application and extension of GA to solve the optimal weights of the attributes under an intuitionistic fuzzy environment.
- Combination of FNIFS theory with GA and TOPSIS further extending the applied range of FNIFS and contributing to its development.

Although several upper-level and domain-specific ontologies exist, few of these express the attributes of the tenderers as FNIFSs. Thus, it is necessary for us to overcome this limitation in a future work and reduce the difficulties of implementing the proposed approach in practice.

## Supporting Information

S1 Code The source code of the software prototype. (ZIP)

S1 Data The whole evaluation information of experts on criteria of all candidate tenderers. (TXT)

S1 File Jar files that need to be imported into the JRE system library. (ZIP)

S2 File Jar files that need to be imported into the JRE system library. (ZIP)

S3 File Jar files that need to be imported into the JRE system library. (ZIP)

S4 File Jar files that need to be imported into the JRE system library. (ZIP)

S1 Text The detailed tenderer information. (TXT)

S2 Text Introduction of the associated source code of the software prototype. (TXT)
