# Supplementary figures and images for: Combined Approach for Government E-Tendering Using GA and TOPSIS with Intuitionistic Fuzzy Information (part 2 of 2)
Source: PLoS One. 2015 Jul 6;10(7):e0130767. doi: 10.1371/journal.pone.0130767 (PMC4493125; doi:10.1371/journal.pone.0130767)

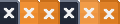

Supplement: S1 Code — (ZIP) [file pone.0130767.s001.zip › Code/JAVA code/WebRoot/js/extjs/resources/ext-theme-access/images/form/clear-trigger.gif]

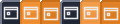

Supplement: S1 Code — (ZIP) [file pone.0130767.s001.zip › Code/JAVA code/WebRoot/js/extjs/resources/ext-theme-access/images/form/date-trigger-rtl.gif]

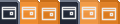

Supplement: S1 Code — (ZIP) [file pone.0130767.s001.zip › Code/JAVA code/WebRoot/js/extjs/resources/ext-theme-access/images/form/date-trigger.gif]

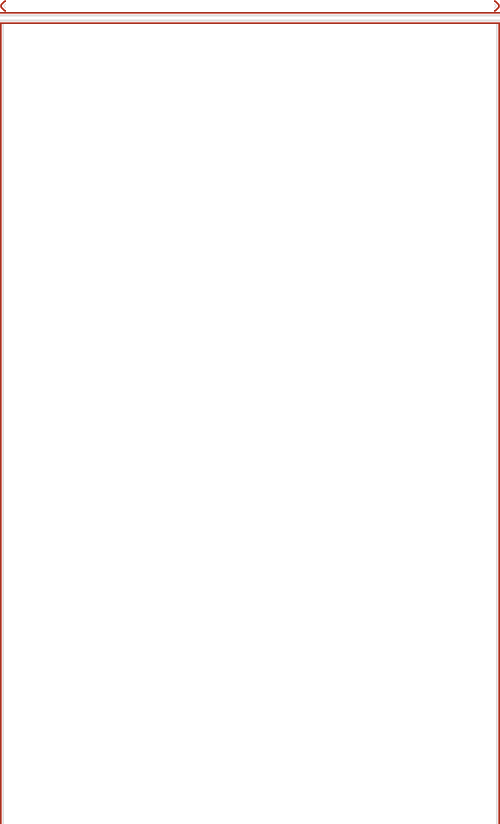

Supplement: S1 Code — (ZIP) [file pone.0130767.s001.zip › Code/JAVA code/WebRoot/js/extjs/resources/ext-theme-access/images/form/error-tip-corners.gif]

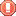

Supplement: S1 Code — (ZIP) [file pone.0130767.s001.zip › Code/JAVA code/WebRoot/js/extjs/resources/ext-theme-access/images/form/exclamation.gif]

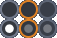

Supplement: S1 Code — (ZIP) [file pone.0130767.s001.zip › Code/JAVA code/WebRoot/js/extjs/resources/ext-theme-access/images/form/radio.gif]

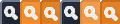

Supplement: S1 Code — (ZIP) [file pone.0130767.s001.zip › Code/JAVA code/WebRoot/js/extjs/resources/ext-theme-access/images/form/search-trigger-rtl.gif]

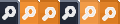

Supplement: S1 Code — (ZIP) [file pone.0130767.s001.zip › Code/JAVA code/WebRoot/js/extjs/resources/ext-theme-access/images/form/search-trigger.gif]

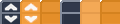

Supplement: S1 Code — (ZIP) [file pone.0130767.s001.zip › Code/JAVA code/WebRoot/js/extjs/resources/ext-theme-access/images/form/spinner-rtl.gif]

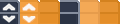

Supplement: S1 Code — (ZIP) [file pone.0130767.s001.zip › Code/JAVA code/WebRoot/js/extjs/resources/ext-theme-access/images/form/spinner-small.gif]

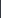

Supplement: S1 Code — (ZIP) [file pone.0130767.s001.zip › Code/JAVA code/WebRoot/js/extjs/resources/ext-theme-access/images/form/text-bg.gif]

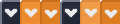

Supplement: S1 Code — (ZIP) [file pone.0130767.s001.zip › Code/JAVA code/WebRoot/js/extjs/resources/ext-theme-access/images/form/trigger-rtl.gif]

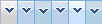

Supplement: S1 Code — (ZIP) [file pone.0130767.s001.zip › Code/JAVA code/WebRoot/js/extjs/resources/ext-theme-access/images/form/trigger-square-rtl.gif]

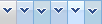

Supplement: S1 Code — (ZIP) [file pone.0130767.s001.zip › Code/JAVA code/WebRoot/js/extjs/resources/ext-theme-access/images/form/trigger-square.gif]
