# Supplementary figures and images for: Combined Approach for Government E-Tendering Using GA and TOPSIS with Intuitionistic Fuzzy Information (part 1 of 2)
Source: PLoS One. 2015 Jul 6;10(7):e0130767. doi: 10.1371/journal.pone.0130767 (PMC4493125; doi:10.1371/journal.pone.0130767)

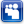

Supplement: S1 Code — (ZIP) [file pone.0130767.s001.zip › Code/JAVA code/WebRoot/image/Logo.png]

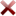

Supplement: S1 Code — (ZIP) [file pone.0130767.s001.zip › Code/JAVA code/WebRoot/image/Toolbar1.png]

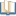

Supplement: S1 Code — (ZIP) [file pone.0130767.s001.zip › Code/JAVA code/WebRoot/image/Toolbar2.png]

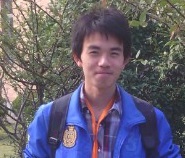

Supplement: S1 Code — (ZIP) [file pone.0130767.s001.zip › Code/JAVA code/WebRoot/image/me1.jpg]

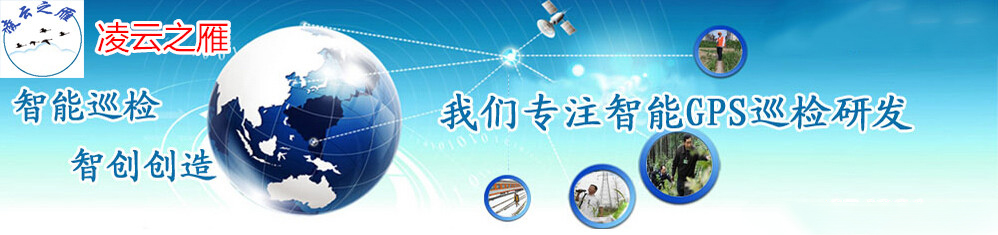

Supplement: S1 Code — (ZIP) [file pone.0130767.s001.zip › Code/JAVA code/WebRoot/image/northimg.jpg]

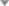

Supplement: S1 Code — (ZIP) [file pone.0130767.s001.zip › Code/JAVA code/WebRoot/js/extjs/resources/ext-theme-access/images/boundlist/trigger-arrow.png]

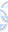

Supplement: S1 Code — (ZIP) [file pone.0130767.s001.zip › Code/JAVA code/WebRoot/js/extjs/resources/ext-theme-access/images/box/corners-blue.gif]

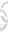

Supplement: S1 Code — (ZIP) [file pone.0130767.s001.zip › Code/JAVA code/WebRoot/js/extjs/resources/ext-theme-access/images/box/corners.gif]

Supplement: S1 Code — (ZIP) [file pone.0130767.s001.zip › Code/JAVA code/WebRoot/js/extjs/resources/ext-theme-access/images/box/l-blue.gif]

Supplement: S1 Code — (ZIP) [file pone.0130767.s001.zip › Code/JAVA code/WebRoot/js/extjs/resources/ext-theme-access/images/box/l.gif]

Supplement: S1 Code — (ZIP) [file pone.0130767.s001.zip › Code/JAVA code/WebRoot/js/extjs/resources/ext-theme-access/images/box/r-blue.gif]

Supplement: S1 Code — (ZIP) [file pone.0130767.s001.zip › Code/JAVA code/WebRoot/js/extjs/resources/ext-theme-access/images/box/r.gif]

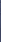

Supplement: S1 Code — (ZIP) [file pone.0130767.s001.zip › Code/JAVA code/WebRoot/js/extjs/resources/ext-theme-access/images/box/tb-blue.gif]

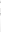

Supplement: S1 Code — (ZIP) [file pone.0130767.s001.zip › Code/JAVA code/WebRoot/js/extjs/resources/ext-theme-access/images/box/tb.gif]

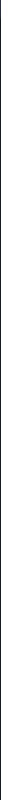

Supplement: S1 Code — (ZIP) [file pone.0130767.s001.zip › Code/JAVA code/WebRoot/js/extjs/resources/ext-theme-access/images/btn/btn-default-large-bg.gif]

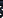

Supplement: S1 Code — (ZIP) [file pone.0130767.s001.zip › Code/JAVA code/WebRoot/js/extjs/resources/ext-theme-access/images/btn/btn-default-large-corners.gif]

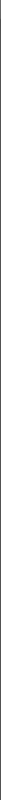

Supplement: S1 Code — (ZIP) [file pone.0130767.s001.zip › Code/JAVA code/WebRoot/js/extjs/resources/ext-theme-access/images/btn/btn-default-large-disabled-bg.gif]

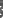

Supplement: S1 Code — (ZIP) [file pone.0130767.s001.zip › Code/JAVA code/WebRoot/js/extjs/resources/ext-theme-access/images/btn/btn-default-large-disabled-corners.gif]

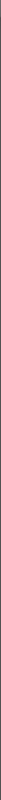

Supplement: S1 Code — (ZIP) [file pone.0130767.s001.zip › Code/JAVA code/WebRoot/js/extjs/resources/ext-theme-access/images/btn/btn-default-large-disabled-fbg.gif]

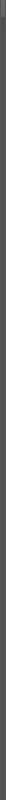

Supplement: S1 Code — (ZIP) [file pone.0130767.s001.zip › Code/JAVA code/WebRoot/js/extjs/resources/ext-theme-access/images/btn/btn-default-large-disabled-sides.gif]

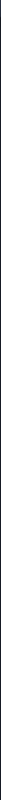

Supplement: S1 Code — (ZIP) [file pone.0130767.s001.zip › Code/JAVA code/WebRoot/js/extjs/resources/ext-theme-access/images/btn/btn-default-large-fbg.gif]

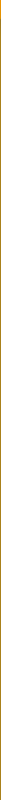

Supplement: S1 Code — (ZIP) [file pone.0130767.s001.zip › Code/JAVA code/WebRoot/js/extjs/resources/ext-theme-access/images/btn/btn-default-large-focus-bg.gif]

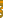

Supplement: S1 Code — (ZIP) [file pone.0130767.s001.zip › Code/JAVA code/WebRoot/js/extjs/resources/ext-theme-access/images/btn/btn-default-large-focus-corners.gif]

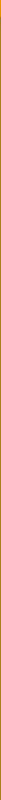

Supplement: S1 Code — (ZIP) [file pone.0130767.s001.zip › Code/JAVA code/WebRoot/js/extjs/resources/ext-theme-access/images/btn/btn-default-large-focus-fbg.gif]

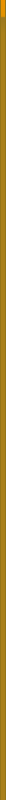

Supplement: S1 Code — (ZIP) [file pone.0130767.s001.zip › Code/JAVA code/WebRoot/js/extjs/resources/ext-theme-access/images/btn/btn-default-large-focus-sides.gif]

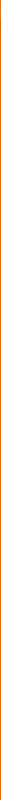

Supplement: S1 Code — (ZIP) [file pone.0130767.s001.zip › Code/JAVA code/WebRoot/js/extjs/resources/ext-theme-access/images/btn/btn-default-large-pressed-bg.gif]

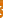

Supplement: S1 Code — (ZIP) [file pone.0130767.s001.zip › Code/JAVA code/WebRoot/js/extjs/resources/ext-theme-access/images/btn/btn-default-large-pressed-corners.gif]

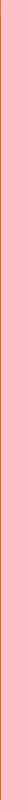

Supplement: S1 Code — (ZIP) [file pone.0130767.s001.zip › Code/JAVA code/WebRoot/js/extjs/resources/ext-theme-access/images/btn/btn-default-large-pressed-fbg.gif]

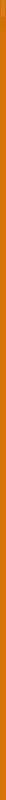

Supplement: S1 Code — (ZIP) [file pone.0130767.s001.zip › Code/JAVA code/WebRoot/js/extjs/resources/ext-theme-access/images/btn/btn-default-large-pressed-sides.gif]

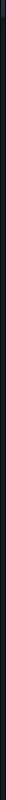

Supplement: S1 Code — (ZIP) [file pone.0130767.s001.zip › Code/JAVA code/WebRoot/js/extjs/resources/ext-theme-access/images/btn/btn-default-large-sides.gif]

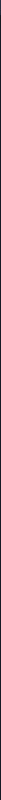

Supplement: S1 Code — (ZIP) [file pone.0130767.s001.zip › Code/JAVA code/WebRoot/js/extjs/resources/ext-theme-access/images/btn/btn-default-medium-bg.gif]

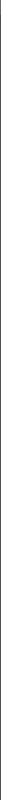

Supplement: S1 Code — (ZIP) [file pone.0130767.s001.zip › Code/JAVA code/WebRoot/js/extjs/resources/ext-theme-access/images/btn/btn-default-medium-disabled-bg.gif]

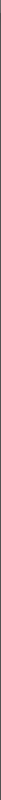

Supplement: S1 Code — (ZIP) [file pone.0130767.s001.zip › Code/JAVA code/WebRoot/js/extjs/resources/ext-theme-access/images/btn/btn-default-medium-disabled-fbg.gif]

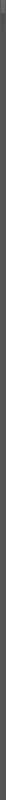

Supplement: S1 Code — (ZIP) [file pone.0130767.s001.zip › Code/JAVA code/WebRoot/js/extjs/resources/ext-theme-access/images/btn/btn-default-medium-disabled-sides.gif]

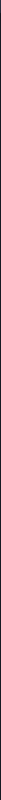

Supplement: S1 Code — (ZIP) [file pone.0130767.s001.zip › Code/JAVA code/WebRoot/js/extjs/resources/ext-theme-access/images/btn/btn-default-medium-fbg.gif]

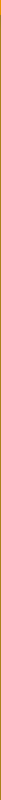

Supplement: S1 Code — (ZIP) [file pone.0130767.s001.zip › Code/JAVA code/WebRoot/js/extjs/resources/ext-theme-access/images/btn/btn-default-medium-focus-bg.gif]

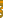

Supplement: S1 Code — (ZIP) [file pone.0130767.s001.zip › Code/JAVA code/WebRoot/js/extjs/resources/ext-theme-access/images/btn/btn-default-medium-focus-corners.gif]

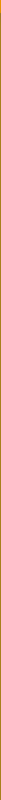

Supplement: S1 Code — (ZIP) [file pone.0130767.s001.zip › Code/JAVA code/WebRoot/js/extjs/resources/ext-theme-access/images/btn/btn-default-medium-focus-fbg.gif]

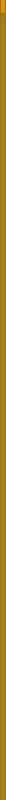

Supplement: S1 Code — (ZIP) [file pone.0130767.s001.zip › Code/JAVA code/WebRoot/js/extjs/resources/ext-theme-access/images/btn/btn-default-medium-focus-sides.gif]

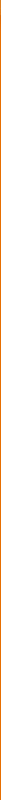

Supplement: S1 Code — (ZIP) [file pone.0130767.s001.zip › Code/JAVA code/WebRoot/js/extjs/resources/ext-theme-access/images/btn/btn-default-medium-pressed-bg.gif]

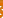

Supplement: S1 Code — (ZIP) [file pone.0130767.s001.zip › Code/JAVA code/WebRoot/js/extjs/resources/ext-theme-access/images/btn/btn-default-medium-pressed-corners.gif]

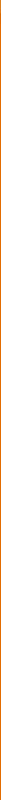

Supplement: S1 Code — (ZIP) [file pone.0130767.s001.zip › Code/JAVA code/WebRoot/js/extjs/resources/ext-theme-access/images/btn/btn-default-medium-pressed-fbg.gif]

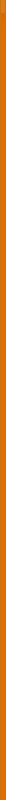

Supplement: S1 Code — (ZIP) [file pone.0130767.s001.zip › Code/JAVA code/WebRoot/js/extjs/resources/ext-theme-access/images/btn/btn-default-medium-pressed-sides.gif]

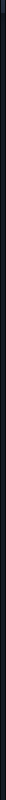

Supplement: S1 Code — (ZIP) [file pone.0130767.s001.zip › Code/JAVA code/WebRoot/js/extjs/resources/ext-theme-access/images/btn/btn-default-medium-sides.gif]

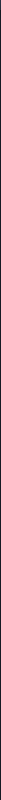

Supplement: S1 Code — (ZIP) [file pone.0130767.s001.zip › Code/JAVA code/WebRoot/js/extjs/resources/ext-theme-access/images/btn/btn-default-small-bg.gif]

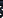

Supplement: S1 Code — (ZIP) [file pone.0130767.s001.zip › Code/JAVA code/WebRoot/js/extjs/resources/ext-theme-access/images/btn/btn-default-small-corners.gif]

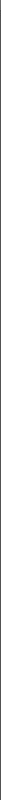

Supplement: S1 Code — (ZIP) [file pone.0130767.s001.zip › Code/JAVA code/WebRoot/js/extjs/resources/ext-theme-access/images/btn/btn-default-small-disabled-bg.gif]

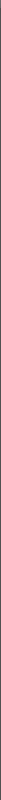

Supplement: S1 Code — (ZIP) [file pone.0130767.s001.zip › Code/JAVA code/WebRoot/js/extjs/resources/ext-theme-access/images/btn/btn-default-small-disabled-fbg.gif]

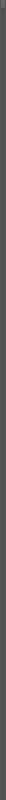

Supplement: S1 Code — (ZIP) [file pone.0130767.s001.zip › Code/JAVA code/WebRoot/js/extjs/resources/ext-theme-access/images/btn/btn-default-small-disabled-sides.gif]

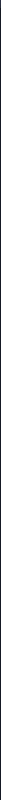

Supplement: S1 Code — (ZIP) [file pone.0130767.s001.zip › Code/JAVA code/WebRoot/js/extjs/resources/ext-theme-access/images/btn/btn-default-small-fbg.gif]

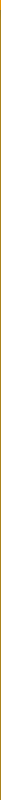

Supplement: S1 Code — (ZIP) [file pone.0130767.s001.zip › Code/JAVA code/WebRoot/js/extjs/resources/ext-theme-access/images/btn/btn-default-small-focus-bg.gif]

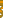

Supplement: S1 Code — (ZIP) [file pone.0130767.s001.zip › Code/JAVA code/WebRoot/js/extjs/resources/ext-theme-access/images/btn/btn-default-small-focus-corners.gif]

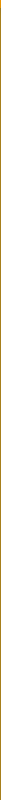

Supplement: S1 Code — (ZIP) [file pone.0130767.s001.zip › Code/JAVA code/WebRoot/js/extjs/resources/ext-theme-access/images/btn/btn-default-small-focus-fbg.gif]

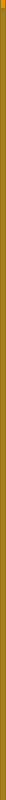

Supplement: S1 Code — (ZIP) [file pone.0130767.s001.zip › Code/JAVA code/WebRoot/js/extjs/resources/ext-theme-access/images/btn/btn-default-small-focus-sides.gif]

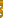

Supplement: S1 Code — (ZIP) [file pone.0130767.s001.zip › Code/JAVA code/WebRoot/js/extjs/resources/ext-theme-access/images/btn/btn-default-small-over-corners.gif]

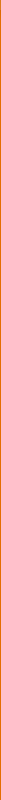

Supplement: S1 Code — (ZIP) [file pone.0130767.s001.zip › Code/JAVA code/WebRoot/js/extjs/resources/ext-theme-access/images/btn/btn-default-small-pressed-bg.gif]

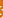

Supplement: S1 Code — (ZIP) [file pone.0130767.s001.zip › Code/JAVA code/WebRoot/js/extjs/resources/ext-theme-access/images/btn/btn-default-small-pressed-corners.gif]

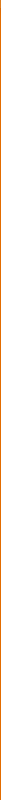

Supplement: S1 Code — (ZIP) [file pone.0130767.s001.zip › Code/JAVA code/WebRoot/js/extjs/resources/ext-theme-access/images/btn/btn-default-small-pressed-fbg.gif]

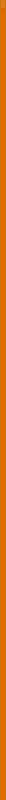

Supplement: S1 Code — (ZIP) [file pone.0130767.s001.zip › Code/JAVA code/WebRoot/js/extjs/resources/ext-theme-access/images/btn/btn-default-small-pressed-sides.gif]

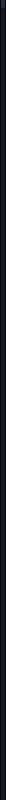

Supplement: S1 Code — (ZIP) [file pone.0130767.s001.zip › Code/JAVA code/WebRoot/js/extjs/resources/ext-theme-access/images/btn/btn-default-small-sides.gif]

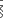

Supplement: S1 Code — (ZIP) [file pone.0130767.s001.zip › Code/JAVA code/WebRoot/js/extjs/resources/ext-theme-access/images/btn/btn-default-toolbar-large-disabled-corners.gif]

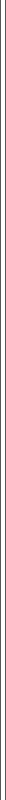

Supplement: S1 Code — (ZIP) [file pone.0130767.s001.zip › Code/JAVA code/WebRoot/js/extjs/resources/ext-theme-access/images/btn/btn-default-toolbar-large-disabled-sides.gif]

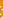

Supplement: S1 Code — (ZIP) [file pone.0130767.s001.zip › Code/JAVA code/WebRoot/js/extjs/resources/ext-theme-access/images/btn/btn-default-toolbar-large-focus-corners.gif]

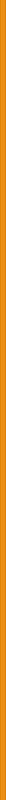

Supplement: S1 Code — (ZIP) [file pone.0130767.s001.zip › Code/JAVA code/WebRoot/js/extjs/resources/ext-theme-access/images/btn/btn-default-toolbar-large-focus-sides.gif]

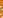

Supplement: S1 Code — (ZIP) [file pone.0130767.s001.zip › Code/JAVA code/WebRoot/js/extjs/resources/ext-theme-access/images/btn/btn-default-toolbar-large-pressed-corners.gif]

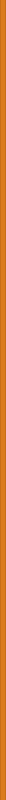

Supplement: S1 Code — (ZIP) [file pone.0130767.s001.zip › Code/JAVA code/WebRoot/js/extjs/resources/ext-theme-access/images/btn/btn-default-toolbar-large-pressed-sides.gif]

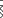

Supplement: S1 Code — (ZIP) [file pone.0130767.s001.zip › Code/JAVA code/WebRoot/js/extjs/resources/ext-theme-access/images/btn/btn-default-toolbar-medium-disabled-corners.gif]

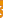

Supplement: S1 Code — (ZIP) [file pone.0130767.s001.zip › Code/JAVA code/WebRoot/js/extjs/resources/ext-theme-access/images/btn/btn-default-toolbar-medium-focus-corners.gif]

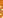

Supplement: S1 Code — (ZIP) [file pone.0130767.s001.zip › Code/JAVA code/WebRoot/js/extjs/resources/ext-theme-access/images/btn/btn-default-toolbar-medium-pressed-corners.gif]

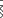

Supplement: S1 Code — (ZIP) [file pone.0130767.s001.zip › Code/JAVA code/WebRoot/js/extjs/resources/ext-theme-access/images/btn/btn-default-toolbar-small-disabled-corners.gif]

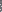

Supplement: S1 Code — (ZIP) [file pone.0130767.s001.zip › Code/JAVA code/WebRoot/js/extjs/resources/ext-theme-access/images/btn-group/btn-group-default-framed-corners.gif]

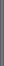

Supplement: S1 Code — (ZIP) [file pone.0130767.s001.zip › Code/JAVA code/WebRoot/js/extjs/resources/ext-theme-access/images/btn-group/btn-group-default-framed-notitle-sides.gif]

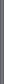

Supplement: S1 Code — (ZIP) [file pone.0130767.s001.zip › Code/JAVA code/WebRoot/js/extjs/resources/ext-theme-access/images/btn-group/btn-group-default-framed-sides.gif]

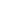

Supplement: S1 Code — (ZIP) [file pone.0130767.s001.zip › Code/JAVA code/WebRoot/js/extjs/resources/ext-theme-access/images/button/arrow.gif]

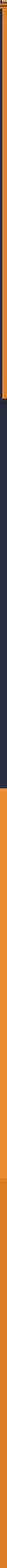

Supplement: S1 Code — (ZIP) [file pone.0130767.s001.zip › Code/JAVA code/WebRoot/js/extjs/resources/ext-theme-access/images/button/btn.gif]

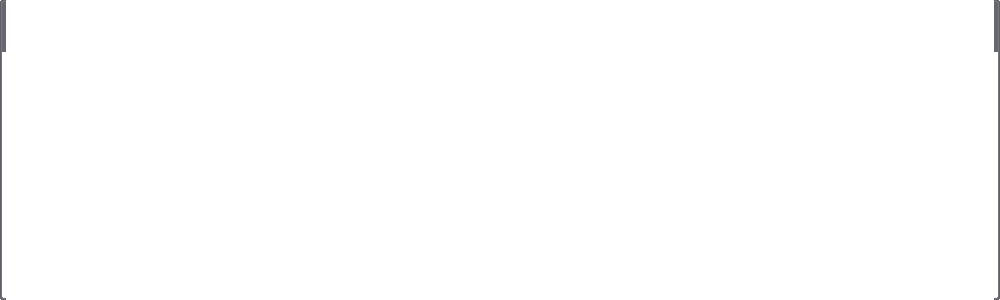

Supplement: S1 Code — (ZIP) [file pone.0130767.s001.zip › Code/JAVA code/WebRoot/js/extjs/resources/ext-theme-access/images/button/group-cs.gif]

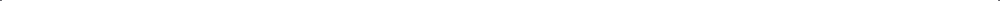

Supplement: S1 Code — (ZIP) [file pone.0130767.s001.zip › Code/JAVA code/WebRoot/js/extjs/resources/ext-theme-access/images/button/group-lr.gif]

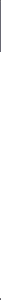

Supplement: S1 Code — (ZIP) [file pone.0130767.s001.zip › Code/JAVA code/WebRoot/js/extjs/resources/ext-theme-access/images/button/group-tb.gif]

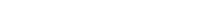

Supplement: S1 Code — (ZIP) [file pone.0130767.s001.zip › Code/JAVA code/WebRoot/js/extjs/resources/ext-theme-access/images/button/s-arrow-b-noline.gif]

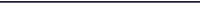

Supplement: S1 Code — (ZIP) [file pone.0130767.s001.zip › Code/JAVA code/WebRoot/js/extjs/resources/ext-theme-access/images/button/s-arrow-b.gif]

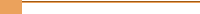

Supplement: S1 Code — (ZIP) [file pone.0130767.s001.zip › Code/JAVA code/WebRoot/js/extjs/resources/ext-theme-access/images/button/s-arrow-bo.gif]

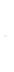

Supplement: S1 Code — (ZIP) [file pone.0130767.s001.zip › Code/JAVA code/WebRoot/js/extjs/resources/ext-theme-access/images/button/s-arrow-light-rtl.gif]

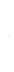

Supplement: S1 Code — (ZIP) [file pone.0130767.s001.zip › Code/JAVA code/WebRoot/js/extjs/resources/ext-theme-access/images/button/s-arrow-light.gif]

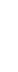

Supplement: S1 Code — (ZIP) [file pone.0130767.s001.zip › Code/JAVA code/WebRoot/js/extjs/resources/ext-theme-access/images/button/s-arrow-noline-rtl.gif]

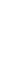

Supplement: S1 Code — (ZIP) [file pone.0130767.s001.zip › Code/JAVA code/WebRoot/js/extjs/resources/ext-theme-access/images/button/s-arrow-noline.gif]

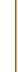

Supplement: S1 Code — (ZIP) [file pone.0130767.s001.zip › Code/JAVA code/WebRoot/js/extjs/resources/ext-theme-access/images/button/s-arrow-o-rtl.gif]

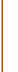

Supplement: S1 Code — (ZIP) [file pone.0130767.s001.zip › Code/JAVA code/WebRoot/js/extjs/resources/ext-theme-access/images/button/s-arrow-o.gif]

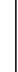

Supplement: S1 Code — (ZIP) [file pone.0130767.s001.zip › Code/JAVA code/WebRoot/js/extjs/resources/ext-theme-access/images/button/s-arrow-rtl.gif]

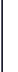

Supplement: S1 Code — (ZIP) [file pone.0130767.s001.zip › Code/JAVA code/WebRoot/js/extjs/resources/ext-theme-access/images/button/s-arrow.gif]

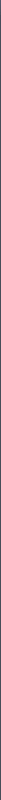

Supplement: S1 Code — (ZIP) [file pone.0130767.s001.zip › Code/JAVA code/WebRoot/js/extjs/resources/ext-theme-access/images/datepicker/datepicker-footer-bg.gif]

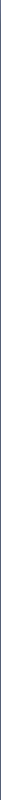

Supplement: S1 Code — (ZIP) [file pone.0130767.s001.zip › Code/JAVA code/WebRoot/js/extjs/resources/ext-theme-access/images/datepicker/datepicker-header-bg.gif]

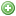

Supplement: S1 Code — (ZIP) [file pone.0130767.s001.zip › Code/JAVA code/WebRoot/js/extjs/resources/ext-theme-access/images/dd/drop-add.gif]

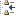

Supplement: S1 Code — (ZIP) [file pone.0130767.s001.zip › Code/JAVA code/WebRoot/js/extjs/resources/ext-theme-access/images/dd/drop-between.gif]

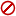

Supplement: S1 Code — (ZIP) [file pone.0130767.s001.zip › Code/JAVA code/WebRoot/js/extjs/resources/ext-theme-access/images/dd/drop-no.gif]

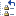

Supplement: S1 Code — (ZIP) [file pone.0130767.s001.zip › Code/JAVA code/WebRoot/js/extjs/resources/ext-theme-access/images/dd/drop-over.gif]

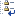

Supplement: S1 Code — (ZIP) [file pone.0130767.s001.zip › Code/JAVA code/WebRoot/js/extjs/resources/ext-theme-access/images/dd/drop-under.gif]

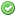

Supplement: S1 Code — (ZIP) [file pone.0130767.s001.zip › Code/JAVA code/WebRoot/js/extjs/resources/ext-theme-access/images/dd/drop-yes.gif]

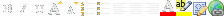

Supplement: S1 Code — (ZIP) [file pone.0130767.s001.zip › Code/JAVA code/WebRoot/js/extjs/resources/ext-theme-access/images/editor/tb-sprite.gif]

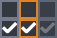

Supplement: S1 Code — (ZIP) [file pone.0130767.s001.zip › Code/JAVA code/WebRoot/js/extjs/resources/ext-theme-access/images/form/checkbox.gif]

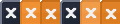

Supplement: S1 Code — (ZIP) [file pone.0130767.s001.zip › Code/JAVA code/WebRoot/js/extjs/resources/ext-theme-access/images/form/clear-trigger-rtl.gif]
